# Effect of therapeutic versus prophylactic anticoagulation therapy on clinical outcomes in COVID-19 patients: a systematic review with an updated meta-analysis

**DOI:** 10.1186/s12959-022-00408-9

**Published:** 2022-08-23

**Authors:** Hong Duo, Yahui Li, Yujie Sun, Liang Wei, Ziqing Wang, Fang Fang, Yuxin Zhong, Jiao Huang, Linjie Luo, Zhiyong Peng, Huaqin Pan

**Affiliations:** 1 Department of Critical Care Medicine, Zhongnan Hospital of Wuhan University, 169 Eastlake Rd., Wuchang, Wuhan, 430071 Hubei province China; 2 The Second Clinical College of Wuhan University, Wuhan, 430071 China; 3 Department of Laboratory Medicine, Zhongnan Hospital of Wuhan University, Wuhan, 430071 China; 4 Clinical Research Center for Critical Care Medicine of Hubei Province, Wuhan, 430071 China; 5 Center for Evidence-Based and Translational Medicine, Zhongnan Hospital of Wuhan University, Wuhan, 430071 China; 6 Department of Experimental Radiation Oncology & Surgical Oncology, The University of Texas MD Anderson Cancer Center, Houston, 77030 USA

**Keywords:** COVID-19, Anticoagulation, Meta-analysis, Randomized clinical trials, Observational studies

## Abstract

**Background:**

Previous studies demonstrate a reduced risk of thrombosis and mortality with anticoagulant treatment in patients with COVID-19 than in those without anticoagulation treatment. However, an open question regarding the efficacy and safety of therapeutic anticoagulation (T-AC) versus a lower dose, prophylaxis anticoagulation (P-AC) in COVID-19 patients is still controversial.

**Methods:**

We systematically reviewed currently available randomized clinical trials (RCTs) and observational studies (OBs) from January 8, 2019, to January 8, 2022, and compared prophylactic and therapeutic anticoagulant treatment in COVID-19 patients. The primary outcomes were risk of mortality, major bleeding, and the secondary outcomes included venous and arterial thromboembolism. Subgroup analysis was also performed between critically ill and non-critically ill patients with COVID-19 and between patients with higher and lower levels of D-dimer. Sensitivity analysis was performed to decrease the bias and the impact of population heterogeneity.

**Results:**

We identified 11 RCTs and 17 OBs fulfilling our inclusion criteria. In the RCTs analyses, there was no statistically significant difference in the relative risk of mortality between COVID-19 patients with T-AC treatment and those treated with P-AC (RR 0.95, 95% CI, 0.78–1.15, *P* = 0.60). Similar results were also found in the OBs analyses (RR 1.21, 95% CI, 0.98–1.49, *P* = 0.08). The pooling meta-analysis using a random-effects model combined with effect sizes showed that in the RCTs and OBs analyses, patients with COVID-19 who received T-AC treatment had a significantly higher relative risk of the major bleeding event than those with P-AC treatment in COVID-19 patients (RCTs: RR 1.76, 95% CI, 1.19–2.62, *P* = 0.005; OBs: RR 2.39, 95% CI, 1.56–3.68, *P* < 0.0001). Compared with P-AC treatment in COVID-19 patients, patients with T-AC treatment significantly reduced the incidence of venous thromboembolism (RR 0.51, 95% CI, 0.39–0.67, *P*<0.00001), but it is not associated with arterial thrombosis events (RR 0.97, 95% CI, 0.66–1.42, *P* = 0.87). The subgroup analysis of OBs shows that the mortality risk significantly reduces in critically ill COVID-19 patients treated with T-AC compared with those with P-AC treatment (RR 0.58, 95% CI, 0.39–0.86, *P* = 0.007), while the mortality risk significantly increases in non-critically ill COVID-19 patients treated with T-AC (RR 1.56, 95% CI, 1.34–1.80, *P* < 0.00001). In addition, T-AC treatment does not reduce the risk of mortality in COVID-19 patients with high d-dimer levels in RCTs. Finally, the overall sensitivity analysis after excluding two RCTs studies remains consistent with the previous results.

**Conclusions:**

In our integrated analysis of included RCTs and OBs, there is no significant difference between the mortality of T-AC and P-AC treatment in unselected patients with COVID-19. T-AC treatment in COVID-19 patients significantly reduced the incidence of venous thromboembolism but showed a higher risk of bleeding than those with P-AC treatment. In addition, P-AC treatment was superior to T-AC treatment in non-critically ill COVID-19 patients, the evidence supporting the necessity for T-AC treatment in critically ill COVID-19 patients came only from OBs.

**Trial registration:**

Protocol registration: The protocol was registered at PROSPERO (CRD42021293294).

**Graphical abstract:**

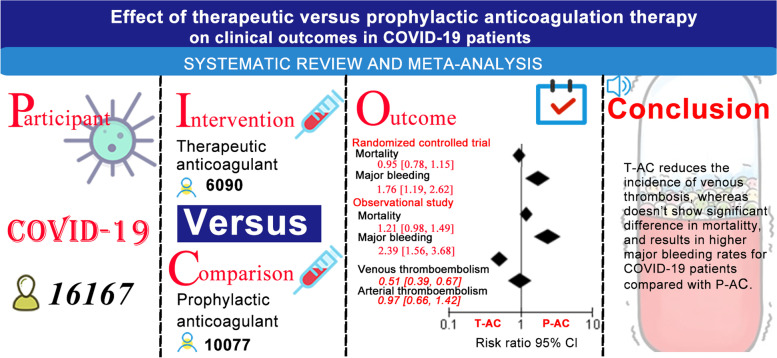

**Supplementary Information:**

The online version contains supplementary material available at 10.1186/s12959-022-00408-9.

## Background

Coronavirus disease 2019 (COVID-19) is an acute infectious disease caused by severe acute respiratory syndrome coronavirus-2 (SARS-CoV-2) [1]. Later, it evolved into a global outbreak and was declared a pandemic by the World Health Organization (WHO) on March 11, 2020. According to the WHO’s weekly epidemiological updates until February 22, 2022, 423,437,674 cases and 5,878,328 deaths have been confirmed, and 10,407,359,583 doses of vaccines have been administered globally [2].

The pathogenesis of COVID-19 infection is associated with severe inflammation and organ damage [3]. It also shows significant pro-thrombotic activity leading to widespread thrombosis and microangiopathy, accompanied by mucus formation in the alveoli of COVID-19 patients [4, 5]. The preference for thrombosis in COVID-19 may be driven by at least two distinct but interrelated processes: (1) the hypercoagulable state leading to microvascular thrombosis and thromboembolism and (2) the direct vascular and endothelial injury leading to in situ microvascular thrombosis [6]. Macrovascular and microvascular thrombosis with inflammatory reactions commonly occurs in hospitalized patients with COVID-19, associated with poor clinical outcomes. Therefore, it is recommended to routinely use anticoagulants in clinical treatment to prevent thrombosis events in COVID-19 patients. Meanwhile, anticoagulants also show anti-inflammatory effects, which can reduce lung damage. However, the choice of anticoagulants, doses, and the standard therapeutic achievement is still inconclusive.

Observational studies (OBs) have shown the benefit of prophylactic anticoagulation for hospitalized COVID-19 patients using various dosing strategies [7, 8]. Anti-thrombotic treatment, including low molecular weight heparin (LMWH) or unfractionated heparin (UFH), has been proposed as a potential therapy for COVID-19 to lower diffuse intravascular clotting activation [9]. However, it is unclear whether prophylaxis anticoagulation (P-AC) or therapeutic anticoagulation (T-AC) have similar efficacy in the clinical outcomes of COVID-19 patients. The data of OBs manifested that both T-AC and P-AC might be associated with lower all-cause mortality compared with no anti-coagulation [10]. However, selection between T-AC and P-AC is still arduous because randomized controlled trials (RCTs) that assess the safety and efficacy of P-AC compared with T-AC found contrasting results [11–14]. It is worth noting that none of these RCTs were powered to test the superiority of individual clinical endpoints, such as all-cause death and major bleeding.

To examine the currently available evidence regarding the effect of anticoagulants in COVID-19 patients, we conducted a systematic review and performed a meta-analysis of published studies (including RCTs and OBs) on the effects of different anticoagulant use (therapeutic-dose versus prophylactic-dose) in the aspects of in-hospital all-cause mortality and other outcomes, providing clinical insights for consideration in the management of COVID-19 patients.

## Methods

This study was performed with adherence to an updated guideline for reporting systematic reviews and meta-analyses (PRISMA) statement [15] (Additional file 1 for PRISMA Checklist). This systematic review protocol was registered with PROSPERO (registration number: CRD42021293294) (Additional file 2 for PROSPERO Protocol).

### Search and select strategy

**Fig. 1:**
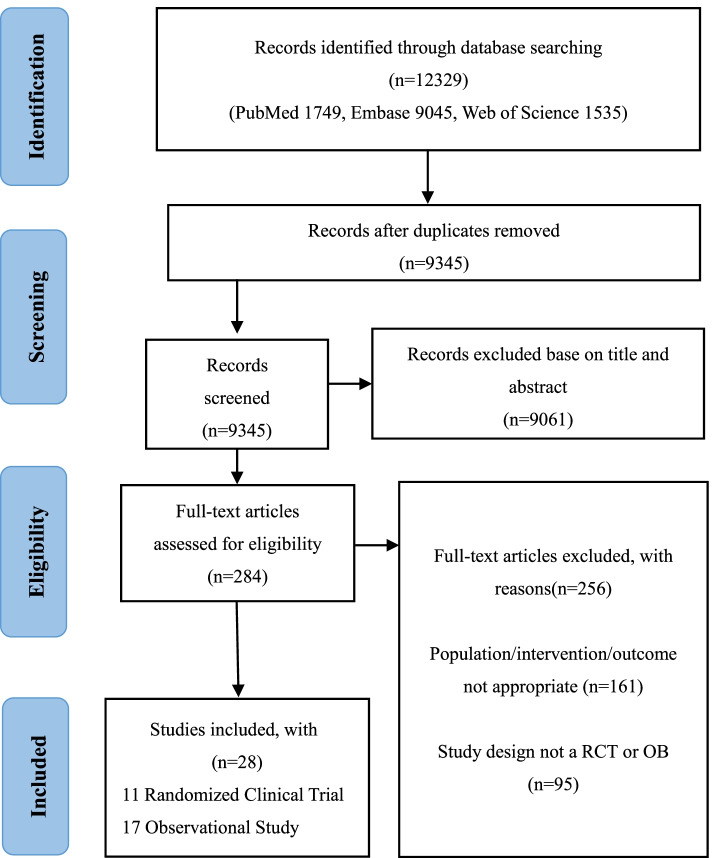
Flow diagram for study selection

Two investigators (HD and LW) independently performed a systematic search of PubMed, Embase, and Web of Science databases using the keywords “COVID-19” and “anticoagulation” and obtained a total of 12,329 articles published from January 8, 2019, to January 8, 2022. To include the relevant literatures as comprehensively as possible, we did not search the databases with applied filters. Restrictions, including age and language, were directly verified by reviewing the full text. After removing duplicates, 9345 articles remained, and 9061 non-relevant articles were further excluded by browsing the titles and abstracts. Regarding the remaining 284 articles to be included in the analysis, we have carefully read the full texts. A flow diagram for study selection is reported in Fig. 1. The search strategies included “COVID-19 OR COVID19 OR SARS-CoV-2” and “anticoagulants OR heparin OR unfractionated heparin” (Additional file 3 for Search Strategies). Meanwhile, we set some restrictions on language (only English), limited sample size (simple size > 10), indicators (mortality, major bleeding), and ages (> 18 years). Our patients included both outpatients and inpatients. Studies were included if they compared T-AC versus P-AC and available data on at least one of our primary outcomes. Case reports, reviews, editorials, commentaries, conference abstracts, protocols, and practice guidelines were excluded (Additional file 4 for PICOS criteria for inclusion and exclusion). We assessed all relevant studies to identify articles for inclusion. We included randomized control studies and observational studies (prospective or retrospective cohort studies) for further analysis.

### Data extraction and risk of Bias assessment

Data extraction was executed independently and systematically by two reviewers (YHL and HD) using a prespecified data extraction form; any controversies were resolved by consensus or arbitrated by a third reviewer (YJS) until disagreement was resolved. Data collection contained study characteristics: first author and year, study design, study population, intervention, the total number of patients, age, gender, BMI, hypertension, cardiovascular disease, diabetes mellitus, chronic kidney disease, chronic pulmonary disease, chronic liver disease, history of smoking, D-dimer, platelet count, mortality, major bleeding, ventilator-free days, major thrombotic events, any bleeding, VTE, and any thrombotic events.

**Fig. 2:**
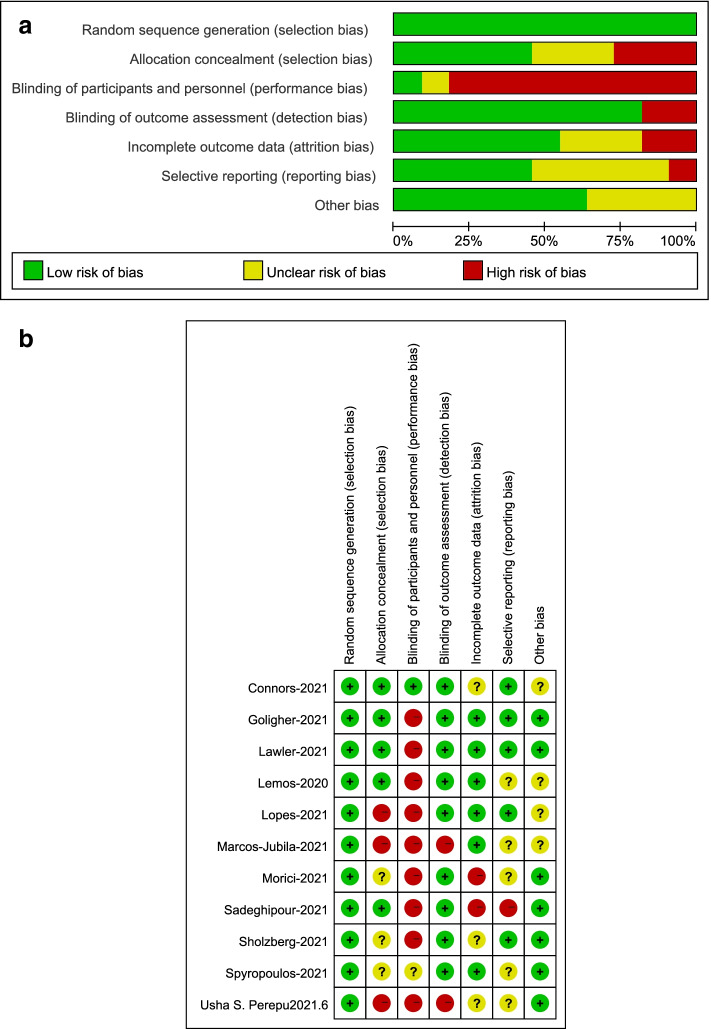
Distribution across studies for each risk of bias item **a.** Risk of bias graph **b.** Risk of bias summary

The methodological quality of the selected articles was respectively assessed by two reviewers (YHL and HD) for the risk of bias. Review Manager 5.4 software (version 1.0 of the Cochrane Risk of Bias Assessment Tool) was used to evaluate the risk of bias of randomized controlled trials [16] with three levels (low risk, unclear risk, and high risk of bias) (Fig. 2). In contrast, the Newcastle-Ottawa Scale was used to assess methodological strength in observational trials [17] with various scores (9 for total score) (Additional file 5).

### Definitions and outcomes

The critically ill patient was defined as severe patients who received respiratory or cardiovascular organ support or other ICU-level care. The non-critically ill patient was defined as ordinary patients who did not receive ICU-level care. It is worth mentioning that each study has different definitions of critically ill and non-critically ill patients, if it’s not violating our above definition principles, we respected the definition of these two types of patients by researchers in each study, and directly extracted the data of the two types of patients from the classified data published in each study. Primary outcomes included: mortality (occurring within the first 90 days due to any causes). Safety outcomes included: major bleeding (conforming to any of the following: type 3, 4, 5 in BARC (Bleeding Academic Research Consortium Definition for Bleeding) [18], type “major” in TIMI (Thrombolysis in Myocardial Infarction bleeding criteria) [18], and type “major bleeding” in ISTH (International Society on Thrombosis and Haemostasis) [19]). Secondary outcomes included venous thromboembolism (VTE) and arterial thromboembolism (ATE). We define VTE as deep vein thrombosis, pulmonary thromboembolism and other venous thromboembolism diseases. We define ATE as stroke, myocardial infarctions, peripheral arterial thromboembolism, and other arterial thromboembolism diseases.

#### Statistical analyses

Meta-analysis was performed using Review Manager 5.4 software (Revman, The Cochrane Collaboration, Oxford, UK). The dichotomous variable was expressed risk ratios (RR), with 95% confidence intervals (CI). As such, pooled analyses were performed by Mantel-Haenszel test with random effects. The clinical heterogeneity among studies was assessed qualitatively. In contrast, the statistical heterogeneity was calculated with the *I*^*2*^ statistic. *I*^*2*^ values > 0%, > 30%, > 50%, and > 75% were considered to indicate low, moderate, substantial, and considerable heterogeneity, respectively [20]. Variability between data is determined by study design, outcomes, and definitions. Subgroup sensitivity analyses were conducted to explore potential sources of heterogeneity. According to our protocol, we selected all-cause mortality as the primary outcome and major bleeding as safety outcome before reading the full texts. Other indicators, such as thrombotic events, are also included as secondary outcomes. To decrease the bias and eliminate the impact of population heterogeneity, we classified the population into critical and non-critical groups and conducted a subgroup analysis to avoid the bias caused by the offset of the total results. ﻿A *p* value < 0.05 was considered significant.

## Results

### Baseline characteristics of included studies

A flow diagram for study selection was shown in Fig. 1**.** We identified a total of 12,329 original literature records through database searching, from inception up to January 8, 2022, which was the date of our final search. After removing the duplicate and screening records based on titles, 284 full-text articles were assessed for eligibility regarding patients’ age, numbers, study design, intervention, and outcomes (Table S2). In the end, 28 studies fulfilled our inclusion criteria, of which 11 were RCTs, and 17 were OBs. These 28 clinical studies involved a total of 16,167 COVID-19 patients (6090 in the T-AC group and 10,077 in the P-AC group). Patients were recruited from the United States, Canada, United Kingdom, Brazil, Mexico, Nepal, Australia, Netherlands, Iran, Italy, Ireland, Saudi Arabia, United Arab Emirates, and Spain.

Among the RCTs, 9 were open-label [11–14, 21–25], and two were blinded [26, 27]. The Cochrane risk assessment for the low risk of blind subjects and intervention providers includes two scenarios: blindness and no blindness with no impact on systematic evaluation. In the case of COVID-19 outbreak, there is no clinical evidence to prove that the advantages and disadvantages of the two treatment strategies will not cause subjective differences due to psychological expectation preference, so it will not affect the systematic evaluation and can be evaluated as low risk. In our assessment of attrition bias, the experimental group or the control group was judged as high risk due to loss of follow-up, withdrawal from non-responder, violation of the treatment plan, or imbalance in the number and reasons for missing outcome data between groups. At the same time, the determination of reporting bias is based on the systematic differences between the results reported in the article and the measured but unreported results, so there is insufficient information to judge low risk or high risk fully. The quality of all included OBs was good, with a score greater than or equal to 5 on the Newcastle-Ottawa Scale (NOS) (Additional file 5). Patients in the experimental intervention group received therapeutic anticoagulation from all the included studies, while patients in the control group received prophylactic anticoagulation. Tables 1 and 2 summarize the study designs of the 28 included trials (more details seen in Additional file 6: Table S1-S2). Of the 11 RCTs, five studies involved critically ill patients [11–13, 21, 27], and six included non-critically ill patients [14, 22–26]. Eight of the 11 RCTs compared therapeutic versus prophylactic doses of anticoagulants, and three trials compared moderate therapeutic versus prophylactic doses of anticoagulants. Seven of the 17 OBs trials involved critically ill patients [29, 31–33, 36, 41, 42], and ten involved non-critically ill patients [8, 28, 30, 34, 35, 37–40, 43]. The outcomes of the 28 clinical studies are summarized in Tables 3 and 4. The risk of bias, including selection bias, performance, detection, attrition, and reporting for all RCTs and individual studies, was assessed (Fig. 2a and b). The funnel plot showed the publication bias (Additional file 7: Fig. S8-S11).

**Table 1:** Characteristics of the 11 RCTs

Author-year	Design	Study population	Intervention	Total, n	Age, median (IQR), year	Male, %	BMI, median (IQR), kg/m^2^
Spyropoulos-2021 [21]	multicenter, active control randomized clinical trial	Hospitalized with COVID-19 (noncritically and critically ill)	Therapeutic (TA): enoxaparin (1 mg/kg BID or 0.5 mg/kg BID)	129	65.8 ± 13.9^a^	52.7	31.2 ± 9.3^a^
			Standard (PA): LMWH (Up to 22,500 IU BID or TID) or enoxaparin (30 mg or 40 mg QD or BID).et	124	67.7 ± 14.1^a^	54.8	29.8 ± 13.6^a^
Lemos-2020 [13]	open-label RCT single-center, phase II	Hospitalized with COVID-19 (and ARDS)	Therapeutic (TA): enoxaparin (1 mg/kg BID or 0.75 mg/kg BID or 1 mg/kg QD)	10	55 ± 10^a^	90	33 ± 8^a^
			Prophylactic (PA): enoxaparin (40 mg QD or 40 mg BID) or UFH (5000 IU TID or 7500 IU TID)	10	58 ± 16^a^	70	34 ± 8^a^
Goligher-2021 [12]	open-label RCT adaptive, multiplatform	Hospitalized with severe COVID-19(critically ill)	Therapeutic (TA): UFH or LMWH	536	60.4 ± 13.1^a^	72.2	30.4 (26.9–36.1)
			Usual-Care (PA): local standard venous thromboprophylaxis	567	61.7 ± 12.5^a^	67.9	30.2 (26.4–34.9)
Lawler-2021 [22]	open-label RCT adaptive, multiplatform	Hospitalized with COVID-19(noncritically ill)	Therapeutic (TA): UFH or LMWH	1181	59.0 ± 14.1^a^	60.4	29.8 (26.3–34.7)
			Usual-Care (PA): local standard venous thromboprophylaxis	1050	58.8 ± 13.9^a^	56.9	30.3 (26.7–34.9)
Connors-2021 [26]	adaptive, randomized, double-blind, placebo-controlled trial	Hospitalized with COVID-19 (symptomatic but clinically stable outpatients)	Therapeutic (TA): apixaban (5 mg BID)	164	52 (47–58)	37.8	31.1 (26.2–35.4)
			Prophylactic (PA): apixaban (2.5 mg BID)	165	55 (46–61)	42.4	29.9 (26.2–34.8)
Marcos-Jubilar-2021 [23]	open-label, multicenter RCT	Hospitalized with COVID-19 (noncritically ill)	Therapeutic (TA): bemiparin (115 IU/kg QD)	32	62.3 ± 12.2^a^	53.1	25.8 (24.0–29.4)
			Standard (PA): bemiparin (3500 IU QD)	33	63.0 ± 13.7^a^	72.7	26.1 (24.1–28.8)
Sholzberg-2021 [24]	Randomized controlled, adaptive, open label clinical trial.	Hospitalized with COVID-19(and elevated d-dimer)	Therapeutic (TA): UFH or LMWH	228	60.4 ± 14.1^a^	53.9	30.3 ± 6.4^a^
			Prophylactic (PA): UFH or LMWH	237	59.6 ± 15.5^a^	59.5	30.2 ± 7.0^a^
Morici-2021 [25]	open-label, multicenter, controlled, randomized trial	Hospitalized with COVID-19 (noncritically ill)	Therapeutic (TA): enoxaparin (40 mg BID)	91	60 (53–73)	61.5	26 (24–28)
			Prophylactic (PA): enoxaparin (40 mg QD)	92	59 (48–72)	64.1	25 (23–28)
Sadeghipour-2021 [27]	Multicenter randomized trial	Admitted to ICU with COVID-19	Intermediate (TA): enoxaparin (1 mg/kg QD)	276	62(51–70.7)	58.7	26.7 (24.4–29.1)
			Standard (PA): enoxaparin (40 mg QD)	286	61(47–71)	57	27.2 (24.3–29.1)
Lopes-2021 [14]	open-label RCT pragmatic	Hospitalized with COVID-19 (stable and unstable)	Therapeutic (TA): rivaroxaban (20 mg or 15 mg QD)	311	56.7 ± 14.1^a^	62	30.3 ± 6.0^a^
			Prophylactic (PA): standard in-hospital enoxaparin or UFH	304	56.5 ± 14.5^a^	58	30.3 ± 6.1^a^
Perepu-2021 [11]	open-label RCT multi-center	Hospitalized with COVID-19 (ICU and/or coagulopathy)	Intermediate (TA): enoxaparin (40 mg QD or 30 mg BID or 40 mg BID)	87	65 (24–86)	54	30.0 (24.7–36.6)
			Standard (PA): enoxaparin (1 mg/kg QD) or0.5 mg/kg BID	86	63.5 (30–85)	58	30.7 (27.2–35.8)

Abbreviations: *BMI* Body mass index, *IQR* Interquartile range, *COVID-19* Coronavirus disease 2019, *TA* Therapeutic anticoagulation, *PA* Prophylactic anticoagulation, *LMWH* Low molecular weight heparin, *UFH* Unfractionated heparin, *ARDS* Advanced remote display system, *QD* Once daily, *BID* Twice daily, *TID* Thrice daily

^a^Reported as mean ± standard deviation

**Table 2:** Characteristics of the 17 OBs

Author-year	Study design	Intervention	Total, n	Age, median (IQR), year	Male, %	BMI, median (IQR), kg/m^2^
Castelnuovo-2021 [28]	Retrospective observational study	Therapeutic (TA): no dose data	418	/	/	/
		Prophylactic (PA): no dose data	983	/	/	/
Elmelhat-2020 [29]	Observational retrospective study	Therapeutic (TA): enoxaparin (1 mg/kg BID)	39	47.0 ± 10.5^a^	74.4	/
		Prophylactic (PA): enoxaparin (40 mg QD)	20	47.7 ± 10.7^a^	90.0	/
Gonzalez-Porras-2021 [30]	Observational study	Therapeutic (TA): enoxaparin (1 mg/kg BID) or bemiparin (115 IU anti-Xa/kg QD)	120	76.3 ± 11.2^a^	59.2	28.8 ± 4.8^a^
		Prophylactic (PA): enoxaparin (40 mg QD) or bemiparin (3500 UQD)	410	71.7 ± 14.1^a^	58.3	28.9 ± 5.3^a^
Hamad-2021 [31]	Retrospective cohort study	Therapeutic (TA): enoxaparin (1 mg/kg BID)	29	59 (51–65)	69.0	28.3 (24.8–32.4)
		High-dose prophylaxis (PA): enoxaparin (40, 50 or 60 mg BID)	17	59 (46–61)	64.7	32.1 (28.4–40)
Helms-2021 [32]	Bi-center cohort study	Therapeutic (TA): LMWH (100 IU/kg/12 h)	71	64 (53–71)	66.2	31 (27–34)
		Prophylactic (PA): LMWH (6000 IU/12 h) or UFH (200 IU/kg/24 h)	108	61 (51–70)	76.9	29 (26–33)
Martinelli-2020 [33]	Observational cohort study	High dose (TA): no dose data	127	60 (51–69)	64.6	27.0 (24.2–30.2)
		Standard (PA): enoxaparin (40 to 60 mg QD)	151	58 (49–66)	65.6	28.1 (25.4–30.2)
Battistoni-2021 [34]	European multicentric cohort study	Full dose (TA): LMWH (40 mg QD)	102	/	/	/
		Prophylactic (PA): LMWH (1 mg/kg BID)	550	/	/	/
Ionescu-2020 [8]	Retrospective, multi-center cohort study	Therapeutic (TA): enoxaparin (1 mg/kg BID or 1.5 mg/kg QD).et	998	68.2 ± 14.6^a^	55.1	30.4 (14.5,73.3)^b^
		Prophylactic (PA): UFH (5000 U BID or TID) or enoxaparin (30–40 mg QD).et	2121	64.4 ± 16.9^a^	46.3	30.4 (12.9, 103.9)^b^
Kaur-2020 [35]	Retrospective, multi-institutional cohort study	Therapeutic (TA): no dose data	381	/	/	/
		Prophylactic (PA): no dose data	652	/	/	/
Canoglu-2020 [36]	Retrospective study	Therapeutic (TA): enoxaparin (1 mg/kg BID)	56	/	/	/
		Prophylactic (PA): enoxaparin (0.5 mg/kg BID)	98	/	/	/
Qin-2021 [37]	A cohort study	Therapeutic (TA): LMWH (100 U/kg BID)	77	/	/	/
		Prophylaxis (PA): LMWH (3000–5000 U QD)	109	/	/	/
Matli-2021 [38]	A propensity matched cohort study	Therapeutic (TA): no dose data	31	62.55 ± 15.80^a^	67.7	/
		Prophylactic (PA): no dose data	51	59.69 ± 17.04^a^	58.8	/
Mennuni-2021 [39]	Observational study	Higher dose (TA): enoxaparin (> 4000 IU QD)	149	70.2 ± 13.0^a^	60.4	/
		Prophylactic (PA): enoxaparin (4000 IU QD)	287	71.2 ± 15.6^a^	55.4	/
Kodama-2020 [40]	A Multi-Center Retrospective Cohort Study	Full dose (TA): no dose data	82	/	/	/
		Prophylactic (PA): no dose data	498	/	/	/
Jonmarker-2020 [41]	Retrospective study	High dose (TA): tinzaparin (≥175 IU/kg QD) or dalteparin (≥200 IU/kg QD)	37	63 (54–70)	31 (83.8)	28.4 (25.1–32.8)
		Low dose (PA): tinzaparin (2500–4500 IU QD) or dalteparin (2500–5000 IU QD)	67	63 (52–71)	59 (88.1)	27.7 (25.5–30.6)
Takayama-2021 [42]	Retrospective historical control study	Therapeutic (TA): UFH (APTT was 1.5–2.5 times as the control)	33	62 (54–74)	87.9	/
		Prophylactic (PA): enoxaparin (40 mg BID)	29	55 (52–65)	86.8	/
Yu-2021 [43]	Retrospective cohort study	Therapeutic (TA): enoxaparin (1 mg/kg BID) or apixaban (≥5 mg BID).et	298	61 (54–72)	63.2	/
		Prophylactic (PA): no dose data	979	62 (50–75)	56	/

Abbreviations: *BMI* Body mass index, *IQR* Interquartile range, *QD* Once daily, *BID* Twice daily, *TID* Thrice daily, *APTT* Activated partial thromboplastin time

^a^Reported as mean ± standard deviation

^b^Reported as median (range)

**Table 3:** Outcomes of the 11 RCTs

Author-year	Intervention	Death, n/total	Major bleeding, n/total	Ventilator-free days, median (IQR)	Major thrombotic events, n (%)	Any bleeding, n (%)	VTE, n (%)	Any thrombotic events, n (%)	Venous thromboembolism	Arterial thromboembolism
Spyropoulos-2021 [21]	TA	25/129	6/129	/	/	/	/	/	12/129	4/129
	PA	31/124	2/124	/	/	/	/	/	33/124	4/124
Lemos-2020 [13]	TA	2/10	0/10	0 (0–11)	2 (20)	2 (20)	/	/	2/10	0/10
	PA	5/10	0/10	15 (6–16)	2 (20)	0 (0)	/	/	2/10	0/10
Goligher-2021 [12]	TA	199/534	20/529	/	34 (6.4)	/	/	38 (7.2)	19/530	23/530
	PA	200/564	13/562	/	58 (10.4)	/	/	62 (11.1)	48/559	22/559
Lawler-2021 [22]	TA	86/1180	22/1180	/	13 (1.1)	/	/	/	16/1180	3/1180
	PA	86/1046	9/1047	/	22 (2.1)	/	/	/	26/1046	5/1046
Connors-2021 [26]	TA	0/164	/	/	/	13 (9.1)	/	/	0/164	0/164
	PA	0/165	/	/	/	9 (6.7)	/	/	0/165	0/165
Marcos-Jubilar-2021 [23]	TA	2/32	0/32	/	0	/	/	/	0/32	0/32
	PA	1/33	0/33	/	2 (6.1)	/	/	/	2/33	0/33
Sholzberg-2021 [24]	TA	4/228	2/228	/	/	/	2 (0.9)	/	2/228	0/228
	PA	18/237	4/237	/	/	/	6 (2.5)	/	6/237	1/237
Morici-2021 [25]	TA	5/91	1/91	/	/	0	0	/	0/91	0/91
	PA	1/92	1/92	/	/	0	6 (6.5)	/	6/92	2/92
Sadeghipour-2021 [27]	TA	119/276	7/276	30 (3–30)	/	17 (6.2)	9 (3.3)	/	9/276	1/276
	PA	117/286	4/286	30 (1–30)	/	9 (3.1)	10 (3.5)	/	10/286	1/286
Lopes-2021 [14]	TA	35/311	10/311	/	23 (7)	36 (12)	11 (4)	/	11/310	14/310/
	PA	23/304	4/304	/	30 (10)	9 (3)	18 (6)	/	18/304	15/304
Perepu-2021 [11]	TA	13/87	2/87	/	/	8 (9)	7 (8)	/	7/87	5/87
	PA	18/86	2/86	/	/	8 (9)	6 (7)	/	6/86	3/86

Abbreviations: *VTE* Venous thromboembolism, *IQR* Interquartile range, *TA* Therapeutic anticoagulation, *PA* Prophylactic anticoagulation

**Table 4:** Outcomes of the 17 OBs

Author-year	Intervention	Death, n/total	Major bleeding, n/total	Major thrombotic events, n (%)	Any bleeding, n (%)	VTE, n (%)	Any thrombotic events, n (%)
Castelnuovo-2021 [28]	TA	62/418	/	/	/	/	/
	PA	114/983	/	/	/	/	/
Elmelhat-2020 [29]	TA	3/39	3/39	/	3 (7.7)	/	/
	PA	0/20	0/20	/	0 (0)	/	/
Gonzalez-Porras-2021 [30]	TA	57/120	6/120	3	/	/	/
	PA	134/410	8/410	7	/	/	/
Hamad-2021 [31]	TA	11/29	6/29	/	/	/	/
	PA	6/17	2/17	/	/	/	/
Helms-2021 [32]	TA	11/71	/	15 (21.1)	/	/	/
	PA	20/108	/	42 (38.9)	/	/	/
Martinelli-2020 [33]	TA	24/127	4/127	/	/	/	/
	PA	50/151	0/151	/	/	/	/
Battistoni-2021 [34]	TA	27/102	9/102	/	/	/	/
	PA	105/550	47/550	/	/	/	/
Ionescu-2020 [8]	TA	236/998	81/998	/	/	/	/
	PA	229/2121	46/2121	/	/	/	/
Kaur-2020 [35]	TA	109/381	/	/	/	/	/
	PA	132/652	/	/	/	/	/
Canoglu-2020 [35]	TA	10/56	/	/	/	/	/
	PA	44/98	/	/	/	/	/
Qin-2021 [37]	TA	25/77)	/	/	/	/	/
	PA	19/109	/	/	/	/	/
Matli-2021 [38]	TA	7 /31	2/31	/	/	/	9 (38.7)
	PA	5/51	2/51	/	/	/	5 (9.8)/
Mennuni-2021 [39]	TA	40/149	1/149	1 (4.8)	/	19 (12.8)	/
	PA	73/287	1/287	1 (1.5)	/	3 (1.1)	/
Kodama-2020 [40]	TA	38/82	7/70	/	/	/	/
	PA	149/498	16/458	/	/	/	/
Jonmarker-2020 [41]	TA	5/37	1/37	/	/	/	/
	PA	26/67	8/67	/	/	/	/
Takayama-2021 [42]	TA	0/33	/	9/29.	/	1 (3.0)	0 (0)
	PA	5/29	/	1/33	/	4(13.8)	0 (0)
Yu-2021 [43]	TA	163/298	41/298	/	/	/	/
	PA	298/979	35/979	/	/	/	/

Abbreviations: *VTE* Venous thromboembolism, *TA* Therapeutic anticoagulation, *PA* Prophylactic anticoagulation

### Primary efficacy outcomes: mortality

**Fig. 3:**
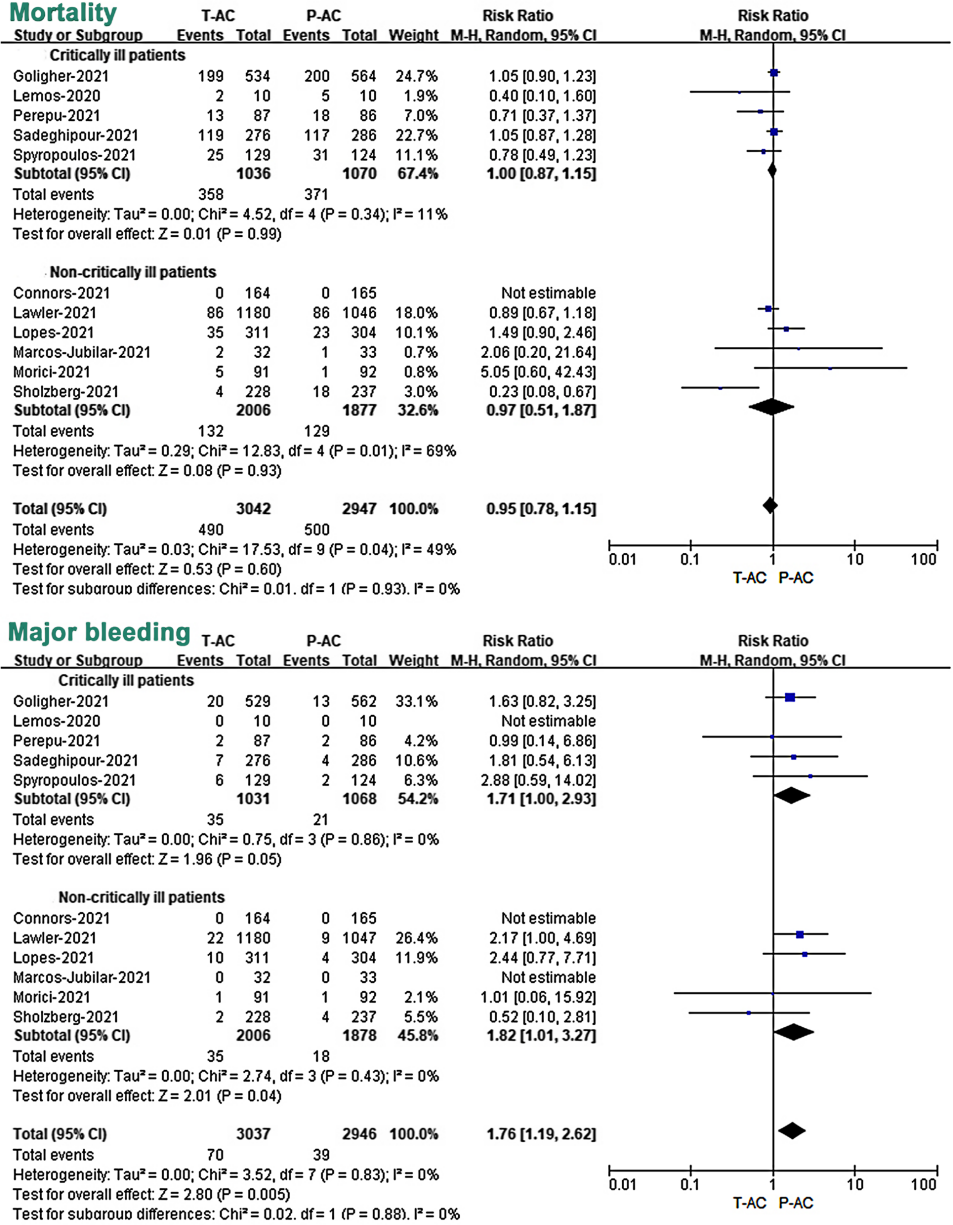
Association of two different dosages of anticoagulant (T-AC vs. P-AC) with primary outcomes (mortality and major bleeding) in pre-specified subgroups (critically vs. non-critically ill patients) of RCTs. T-AC = therapeutic anticoagulation; P-AC = prophylactic anticoagulation

**Fig. 4:**
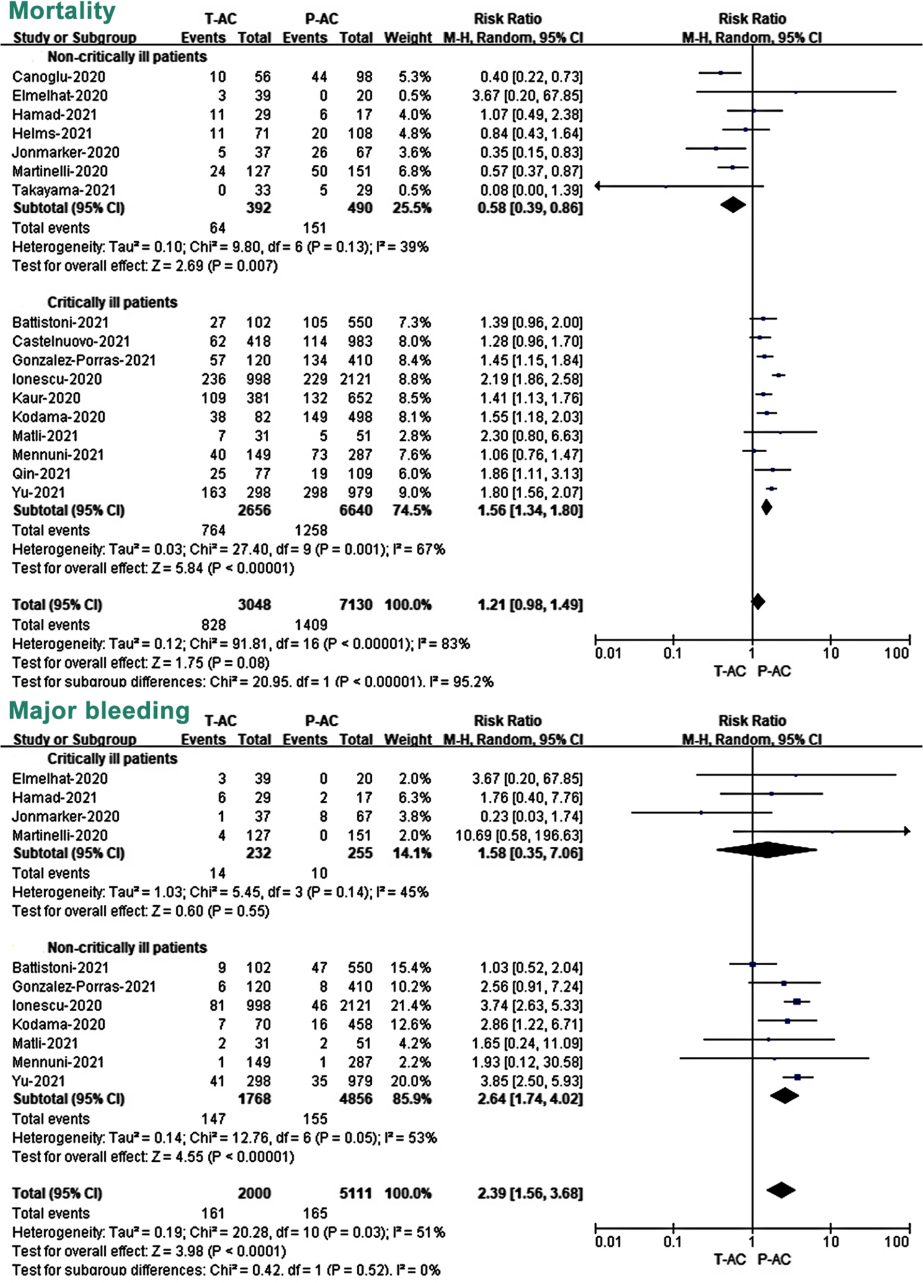
Association of two different dosages of anticoagulant (T-AC vs. P-AC) with primary outcomes (mortality and major bleeding) in pre-specified subgroups (critically vs. non-critically ill patients) of OBs. T-AC = therapeutic anticoagulation; P-AC = prophylactic anticoagulation

All 28 included studies have reported mortality as an outcome measure. Overall, the 11 RCTs reported an overall mortality rate of 16.1% (490/3042) and 17.0% (500/2947) in patients treated with T-AC and P-AC, respectively (Fig. 3; Additional file 7: Fig. S1). In the 17 OBs, the mortality of the two groups was 27.2% (828/3048) and 19.8% (1409/7130), respectively (Fig. 4; Additional file 7: Fig. S2). Our meta-analysis of included RCTs showed that T-AC resulted in a non-significant reduction in overall mortality compared to P-AC (RR 0.95, 95% CI, 0.78–1.15, *P* = 0.60). (Fig. 3; Additional file 7: Fig. S1). However, our meta-analysis of included OBs showed that T-AC had a 21% increase in overall mortality compared to P-AC, although this trend towards increased mortality in the T-AC group did not reach a statistical difference (RR 1.21, 95% CI, 0.98–1.49, *P* = 0.08) (Fig. 4; Additional file 7: Fig. S2).

### Safety outcomes: major bleeding

The major bleeding event was reported in all included RCTs and 11 of the 17 OBs. Overall, in 11 RCTs, the incidence of major bleeding in COVID-19 patients treated with T-AC and P-AC was 2.3% (70/3037) and 1.3% (39/2946), respectively (Fig. 3; Additional file 7: Fig. S3), while in the 11 OBs studies, the incidence of major bleeding in the two groups was 8.1% (161/2000) and 3.2% (165/5111), respectively (Fig. 4; Additional file 7: Fig. S4). Our meta-analysis of included RCTs showed that T-AC resulted in a significant increase in major bleeding compared to P-AC. (RR 1.76, 95% CI, 1.19–2.62, *P* = 0.005) (Fig. 3; Additional file 7: Fig. S3). Similar results were also shown in the OBs (RR 2.39, 95% CI, 1.56–3.68, *P* < 0.0001) (Fig. 4; Additional file 7: Fig. S4).

### Secondary outcomes: venous thromboembolism and arterial thromboembolism

**Fig. 5:**
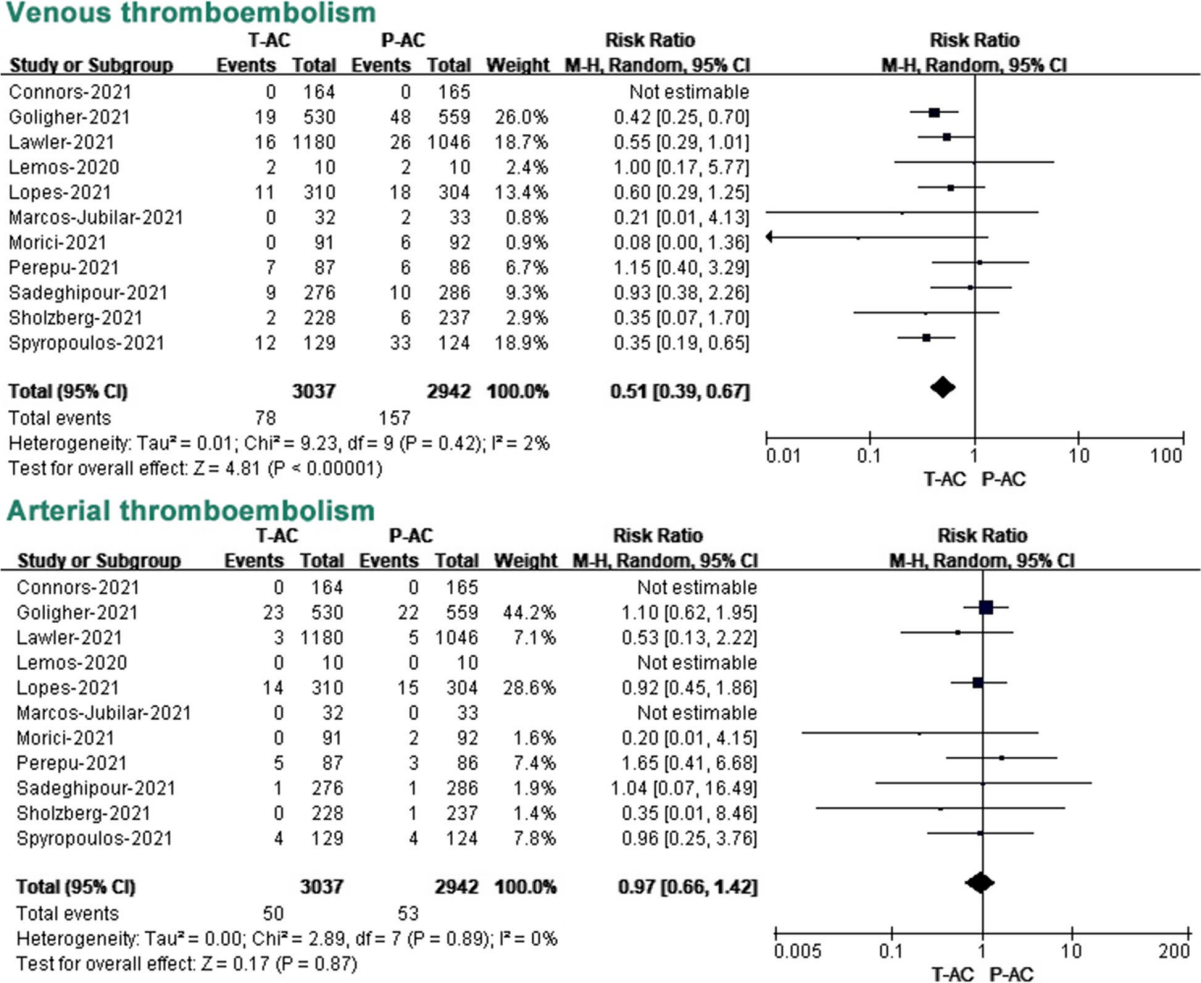
Association of two different dosages of anticoagulant (T-AC vs. P-AC) with secondary outcomes (venous and arterial thromboembolism) in RCTs. T-AC = therapeutic anticoagulation; P-AC = prophylactic anticoagulation

We next studied whether the incidence of arteriovenous thromboembolic events was differentially reduced in COVID-19 patients with two doses of anticoagulation treatment. Due to limited data on thromboembolic events in the OBs, we analyzed 11 RCTs with all reported arteriovenous thromboembolic events, with little heterogeneity among these studies. The incidence of venous thrombosis in COVID-19 patients treated with T-AC and P-AC was 2.6% (78/3037) and 5.3% (157/2942), respectively, while the incidence of arterial thrombosis was 1.6% (50/3037) and 1.8% (53/2942), respectively. Our meta-analysis of included OBs showed that VTE was reduced by 49% in T-AC compared to P-AC. (RR 0.51, 95% CI, 0.39–0.67, *P* < 0.00001, *I*^*2*^ = 2%). However, there was a non-significant difference in ATE between T-AC and P-AC. (RR 0.97, 95% CI, 0.66–1.42, *P* = 0.87, *I*^*2*^ = 0%) (Fig. 5).

### Subgroup analysis: critically ill versus non-critically ill patients

To further study the effect of severity in outcomes of patients with two doses of anticoagulation treatment, we divided the included population into critically ill and non-critically ill patients and performed meta-analysis subgroup analyses on mortality, major bleeding, and thromboembolic events in the two studies, respectively. Our subgroup analysis of included RCTs showed that there was a non-significant difference in mortality between T-AC and P-AC in both critically ill (RR 1.00, 95% CI, 0.87–1.15, *P* = 0.99) and non-critically ill patients (RR 0.97, 95% CI, 0.51–1.87, *P* = 0.93) (Fig. 3). However, our subgroup analysis of included RCTs showed that T-AC resulted in a significant increase in major bleeding compared to P-AC in both critically ill patients (RR 1.71, 95% CI, 1.00–2.93, *P* = 0.05) and in non-critically ill patients (RR 1.82, 95% CI, 1.01–3.27, *P* = 0.04) (Fig. 3).

Interestingly, our subgroup analysis of included OBs showed that T-AC had a significant reduction in mortality compared to P-AC in critically ill patients. (RR 0.58, 95% CI, 0.39–0.86, *P* = 0.007). On the contrary, T-AC had a significant increase in mortality compared to P-AC in non-critically ill patients. (RR 1.56, 95% CI, 1.34–1.80, *P* < 0.00001). A random-effects meta-analysis model pooled mortality risk, and effect sizes showed opposite outcomes in the two groups (Fig. 4). Our subgroup analysis of included OBs shows T-AC had a significant increase in major bleeding compared to P-AC in non-critically ill patients. (RR 2.64, 95% CI, 1.74–4.02, *P* < 0.00001), while it did not show a statistically significant difference in critically ill patients (RR 1.58, 95% CI, 0.35–7.06, *P* = 0.55) (Fig. 4). In addition, our subgroup analysis of included RCTs shows T-AC had a significant reduction in VTE compared to P-AC in both critically ill patients (RR 0.57, 95% CI, 0.35–0.91, *P* = 0.02) and non-critically ill patients (RR 0.51, 95% CI, 0.33–0.79, *P* = 0.003). However, regarding ATE, our subgroup analysis of included RCTs showed no statistical difference between P-AC and T-AC in both critically ill patients (RR 1.14, 95% CI, 0.70–1.85, *P* = 0.61) and non-critically ill patients (RR 0.75, 95% CI, 0.41–1.39, *P* = 0.36). (Additional file 7: Fig. S5).

### Subgroup analysis (mortality): high d-dimer levels

Five RCT clinical trials (Lawler [22], Marcos-Jubllar [23], Perepu [11], Sadeghipour [27], Sholzberg [24]) included COVID-19 patients with high d-dimer levels, so we performed a subgroup analysis of mortality risk. The results showed T-AC had a non-significant reduction in mortality compared to P-AC in high d-dimer levels patients. (RR 0.86, 95% CI, 0.64–1.17, *P* = 0.34) (Additional file 7: Fig. S6).

### Sensitivity analysis

**Fig. 6:**
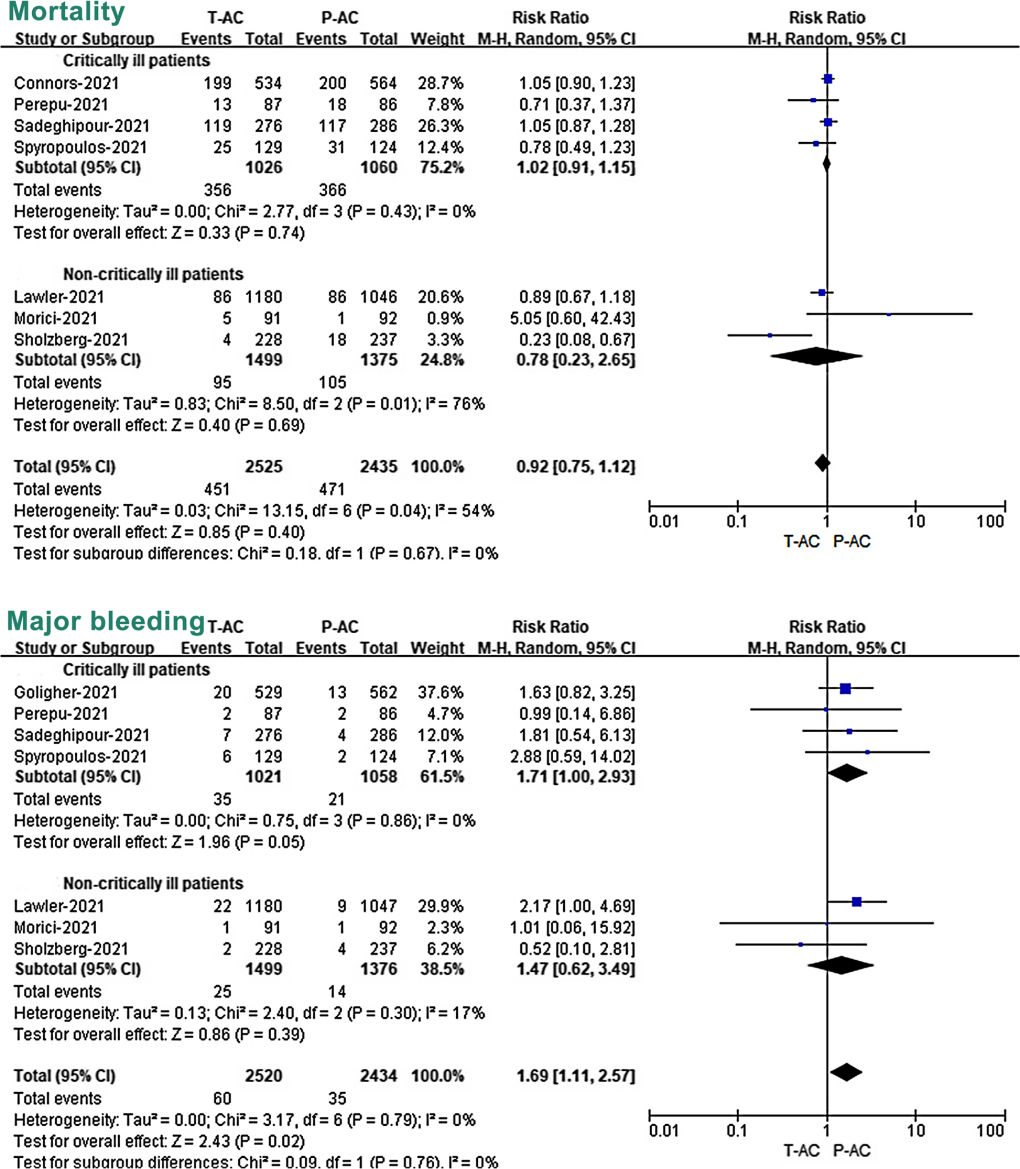
Sensitivity analysis of primary outcomes (mortality and major bleeding) by excluding 4 studies with high heterogeneity. T-AC = therapeutic anticoagulation; P-AC = prophylactic anticoagulation

In RCTs and OBs, sensitivity analyses under random-effects and fixed-effects models showed no significant differences between the prophylactic and therapeutic doses of anticoagulation treatment in the results of overall and subgroup analyses (Fig. 6; Additional file 8: Table S1). However, the mortality in OBs showed a statistically significant difference (RR 1.48, 95% CI, 1.37–1.59, *P* < 0.00001) (Additional file 7: Fig. S7). To further explore and address the heterogeneity, we excluded data that may have a significant impact on the combined effect size according to the following criteria. After excluding rivaroxaban, apixaban, and two small-sized data, the combined effect sizes obtained by our analysis (Additional file 8: Table S2) were not different from those before (Fig. 6).

## Discussion

This comprehensive meta-analysis includes 11 RCTs (5989 patients) and 17 OBs (10,178 patients), which compared T-AC versus P-AC treatment in a total of 16,167 COVID-19 patients and analyzed all-cause mortality, events of major bleeding, thrombosis, and outcomes of subgroups. The results showed that: (1) in terms of RCTs, T-AC was not associated with a lower risk of all-cause mortality in the COVID-19 patients, compared to P-AC, and the clinical benefit to patients with COVID-19 is unclear. However, the subgroup analysis of OBs shows that the mortality risk significantly reduces in critically ill COVID-19 patients treated with T-AC compared with those with P-AC treatment. In contrast, the mortality risk significantly increases in non-critically ill COVID-19 patients treated with T-AC. (2) In RCTs and OBs analyses, T-AC treatment has a significantly higher risk of major bleeding than P-AC treatment in COVID-19 patients. (3) Compared with P-AC treatment in COVID-19 patients, patients with T-AC treatment significantly reduce the incidence of venous thromboembolism, but it is not associated with arterial thrombosis events. (4) T-AC treatment does not reduce mortality risk in COVID-19 patients with high d-dimer levels in RCTs, consistent with overall outcome measures. (5) The overall sensitivity analysis results after excluding RCTs data remain consistent with the previous results.

Previous clinical studies of patients with COVID-19 demonstrate that anticoagulant treatment reduces the risk of mortality and thrombosis compared to that without anticoagulation, which generally benefits patients [44]. Similarly, two previous studies show benefits in patients receiving anticoagulation, which is associated with increased survival compared with patients without anticoagulation treatment [7, 45]. However, there is an open question regarding anticoagulation doses treatment in COVID-19 patients, and whether therapeutic doses of anticoagulants are more effective than low-dose anticoagulation for prophylaxis is still controversial.

In our meta-analysis, the COVID-19 patients treated with T-AC compared with P-AC treatment did not reduce mortality risk. However, in the OBs, T-AC treatment in COVID-19 patients significantly reduces the mortality in critically ill patients compared with the patients with P-AC treatment. Surprisingly, in non-critically ill COVID-19 patients, T-AC treatment can increase mortality compared with the patients with P-AC treatment. Similar results are reported in another meta-analysis of observational studies [45]. In addition, recently published meta-analyses show no survival benefit with higher doses of anticoagulants in COVID-19 patients, and they both increase the risk of major bleeding [46–48]. The results demonstrate that therapeutic doses increase bleeding events, while prophylactic doses decrease the risk of bleeding in COVID-19 patients. It is well known that exposure to high doses of anticoagulants can lead to major bleeding events, often with fatal consequences [49–51].

In our meta-analysis regarding arterial and venous thrombosis events, T-AC treatment in COVID-19 patients significantly reduces the risk of venous thrombosis compared with P-AC treatment and increases the risk of major bleeding. However, the risk of arterial thrombosis did not decrease in COVID-19 patients treated with T-AC, consistent with previous studies [46–48]. The possible mechanisms for these results may be related to different pathogenesis of arterial and venous thrombosis. Venous thrombosis can be triggered by blood stasis, hypercoagulability, and endothelial dysfunction and occurs most commonly in the valve pockets of large veins [52]. However, arterial thrombosis, caused by atherosclerosis, is mainly formed by the aggregation of platelets [53]. The prophylaxis of arterial thrombosis is usually benefited from antiplatelet therapy. Whereas recent studies from three larger, open-label, randomized controlled trials do not support the addition of antiplatelet treatment to ﻿prevent progressive thromboinflammatory complications in hospitalized COVID-19 patients [54–56]. D-dimer levels were identified to be associated with vascular thrombosis and a poor clinical outcome in critical illness, which might suggest a strategy of d-dimer-guided anticoagulation [57]. Compared with P-AC treatment in COVID-19 patients, T-AC treatment does not reduce mortality risk in patients with high d-dimer levels (Additional file 7: Fig. S6). Its efficacy needs to be further studied.

Moreover, two included studies used rivaroxaban and apixaban as anticoagulant agents: Lopes-2021 [14] and Connors-2021 [26]. Both rivaroxaban and apixaban are orally available, direct factor Xa inhibitors with a different mechanism of action than heparin. They are small molecules with a distribution volume exceeding heparin [58, 59], potentially allowing them to better access lung tissue to prevent alveolar thrombosis. Rivaroxaban and apixaban are used in outpatient settings because they are limited to non-critically ill COVID-19 patients. However, it is still unclear whether this diverse mechanism reflects a clinical difference between anticoagulant treatment with rivaroxaban or apixaban and LMWH/UFH in patients with COVID-19. In addition, due to a small sample size of two sets of data (Lemos-2020 [13], Marcos-Jubilar-2021 [23]), we excluded studies of rivaroxaban and apixaban treatment in COVID-19 patients from the subsequent sensitivity analysis to decrease type II errors. The results from the sensitivity analyses after exclusion are consistent with previous mortality and major bleeding results. Sensitivity analyses for OBs are not performed due to insufficient baseline characteristics and a lack of data integrity.

In addition, UFH prolongs clotting time by enhancing or activating antithrombin III (AT-III) and anticoagulant factor Xa. Compared with UFH, LMWH exerts an anticoagulant effect by primarily enhancing and activating anticoagulant factor Xa [9]. There was no significant difference in efficacy between UFH and LMWH [9]. LMWH has the advantages of a robust anticoagulant effect, low risk of major bleeding, and low probability of inducing heparin-related thrombocytopenia (HIT), making it more preferred in the clinic. However, in critically ill patients, especially those with renal insufficiency or the elderly, LMWH tends to cause drug accumulation, leading to an increased risk of bleeding in these patients [60, 61]. In current available studies, many populations were unclassified, resulting in increased heterogeneity of the study populations and possibly inconsistent results between different uses of UFH and LMWH.

The clinical trial of “Therapeutic Anticoagulation versus Standard Care as a Rapid Response to the COVID-19 Pandemic” (RAPID) was to determine if therapeutic heparin is superior to prophylactic heparin in moderately ill patients with COVID-19 [24]. Their results showed no significant reduction in the primary outcome of death, mechanical ventilation, or length of ICU stay with therapeutic heparin. However, therapeutic heparin was associated with a significant decrease in all-cause mortality and a reduced risk of major bleeding in the patients with COVID-19. The results from the RAPID trial suggest that therapeutic heparin was beneficial to moderately ill patients with COVID-19 who were admitted to hospital wards [24]. A large observational study of 3119 patients with COVID-19 showed that both prophylactic and therapeutic doses reduced mortality in COVID-19 patients with hypercoagulable states [8]. In addition, COVID-19 patients who received the therapeutic dose had a higher survival probability than those with the prophylactic dose treatment. But in critically ill patients, there was an increased probability of major bleeding [8]. Notably, the benefit of high-dose anticoagulants is unclear due to active or potential bleeding complications, low baseline hemoglobin or platelet counts, and physician practice choices.

Based on the above interpretation and analysis, compared without anticoagulants treatment, COVID-19 patients prone to hypercoagulability have increased clinical benefits of anticoagulation treatment. However, the choice of dose between T-AC and P-AC is still controversial. In our integrated analysis of included RCTs and OBs, the evidence supporting the necessity for T-AC treatment in critically ill COVID-19 patients came only from OBs. Still, close monitoring of the associated bleeding risk is also required. A previous study showed a hypercoagulable state in the COVID-19 presents during the early stage of infection [62]. Therefore, prompt anticoagulation treatment may prevent the disease from progressing to severe forms in COVID-19 patients who may present with a disseminated intravascular coagulopathy (DIC)-like state [63], thus preventing an increased risk of major bleeding.

Meanwhile, prophylactic anticoagulants may be the most beneficial option in non-critically ill patients, reducing the incidence of major bleeding and fatal events. Routine use of therapeutic doses of anticoagulants beyond the standard prophylactic dose is not suggested in non-critically ill patients with COVID-19. Even though thromboprophylaxis does not reduce mortality in patients with acute illness [64], it can be used to prevent venous thrombotic events in patients without indications for anticoagulation as a guideline-recommended [65]. Thromboprophylaxis reduces the risk of thrombosis in patients with risk factors but not the risk of death [64]. Therefore, for patients without anticoagulation indications, the benefits of venous thrombosis prophylaxis outweigh the risks, which may be clinically beneficial. In COVID-19 patients with a hypercoagulable state, the prophylactic dose may be less effective than the therapeutic dose for preventing venous thrombosis. In addition, another concern is the lack of guidance for the anticoagulation treatment window timing and symptom onset time, even though the average time window for symptoms of COVID-19 patients is about one-week [66]. Therefore, further clinical trials are needed to confirm the effectiveness of the study on the time window of coagulation to prevent thrombosis in patients with COVID-19.

### Strengths and limitations

The current comprehensive meta-analysis study incorporated all relevant RCTs and OBs and analyzed more studies and patients with COVID-19 than previous studies, which only included RCTs [46]. In RCTs, we did not find a statistical difference between the mortality of T-AC and P-AC treatment in patients with COVID-19. However, data analysis from OBs demonstrated that T-AC significantly increased the survival of critically ill patients with COVID-19, but not the non-critically ill patients. In both RCTs and OBs, T-AC treatment in COVID-19 patients showed a decreased risk of venous thromboembolism but increased the risk of bleeding compared to P-AC treatment.

Contrasting with merely pooling RCTs, we discovered some controversies about the conclusion of the meta-analysis. Observational studies play an essential role in guiding patient care, making medical decisions, and providing evidence among representative patients with varying severity. Nowadays, policymakers increasingly require much real-world data such as electronic medical records to conduct observational studies on the medical evidence and clinical practice [67]. RCTs provide the primary evidence for regulatory decisions. Still, as the methods of real-world research mature, there will be more opportunities to supplement the limited aspects of RCTs using real-world research [68].

We agree that selection bias and other confounding factors are usually caused by imperfect experimental design in observational studies. However, our analysis and judgment of experimental results are not only based on the confounding factors and biases of observational studies, but also observational studies have the advantage of helping us to clarify the results better. Nevertheless, in the context of the COVID-19 pandemic, OBs came before a clear rationale for the choice between T-AC and P-AC has been produced by rigorous RCTs. For this reason, data from OBs might be influenced or biased by the different patient severity which might have affected clinician decisions on treatment and dose assignment due to hypothetical benefits or personal expectations. This may explain the observed discrepancy between early OBs and subsequent RCTs in terms of mortality in critically and non-critically ill patients. Of course, future high-quality large RCTs and well-conducted OBs based on new real-world data coming after RCTs results could eventually clarify the best anticoagulation therapy in COVID-19 patients.

However, several limitations to our study should be noted. First, due to the limitations of baseline characteristics, we did not perform detailed subgroup analyses, and all analyses were classified into severe and non-severe cases according to clinical conditions. In addition, the quality of the included RCTs varied, and all but two trials had an open-label design, which may lead to bias in the determination of thrombotic and bleeding events. Detailed sensitivity analyses for OBs were not performed due to a lack of information. Second, the sample sizes of two studies were too small with inadequate representation, and two used rivaroxaban and apixaban as anticoagulation treatment in patients with COVID-19. This may lead to increased heterogeneity and statistical error in type II. But we performed a sensitivity analysis later and excluded them. Thirdly, the population definitions used in the studies are different, such as critically ill and non-critically ill patients, which cannot be summarized uniformly, resulting in biased population classification. In addition, there was no standardization of prophylactic and therapeutic treatment strategies, and definitions of primary and secondary outcomes were inconsistent. Therefore, our results should be considered carefully, as possible confounding cannot be completely ruled out. Fourth, the observation methods and time of outcome indicators, such as major bleeding, were inconsistent in different studies. There was heterogeneity among study populations, settings, experimental designs, interventions, and detection methods. Finally, there was one study that used direct oral anticoagulants [25] as its therapeutic anticoagulation, and two other studies used rivaroxaban or bemisaban.

## Conclusions

In our integrated analysis of included RCTs and OBs, there is no significant difference between the mortality of T-AC and P-AC treatment in unselected patients with COVID-19. T-AC treatment showed a higher risk of bleeding than the COVID-19 patients with P-AC treatment. Compared to P-AC, T-AC treatment was associated with a significantly decreased risk of venous thromboembolism in the patients with COVID-19. However, there was no association with arterial thromboembolism. In addition, P-AC treatment was superior to T-AC treatment in non-critically ill COVID-19 patients, and the evidence supporting the necessity for T-AC treatment in critically ill COVID-19 patients came only from OBs.

## Supplementary Information

**Additional file 1.** PRISMA 2020 Checklist.

**Additional file 2.** PROSPERO Protocol.

**Additional file 3.** Search Strategies.

**Additional file 4.** PICOs criteria for inclusion and exclusion of studies.

**Additional file 5.** Newcastle-Ottawa Scale.

**Additional file 6: Table S1.** Patient Baseline Characteristics in the 11 RCTs. **Table S2.** Patient Baseline Characteristics in the 17 OBs.

**Additional file 7: Fig. S1.** Mortality of therapeutic anti-coagulation vs. prophylactic anticoagulation in RCTs. **Fig. S2.** Major bleeding of therapeutic anti-coagulation vs. prophylactic anticoagulation in RCTs. **Fig. S3.** Mortality of therapeutic anti-coagulation vs. prophylactic anticoagulation in OBs. **Fig. S4.** Major bleeding of therapeutic anti-coagulation vs. prophylactic anticoagulation in OBs. **Fig. S5.** Subgroup analysis of thromboembolism among critically or non-critically ill patients in OBs. **Fig. S6.** Subgroup analysis of mortality in studies of patients with high d-dimer level. **Fig. S7.** Sensitivity analysis of mortality in OBs. **Fig. S8.** Funnel plot: Mortality of RCTs. **Fig. S9.** Funnel plot: Major bleeding of RCTs. **Fig. S10.** Funnel plot: Mortality of OBs. **Fig. S11.** Funnel plot: Major bleeding of OBs.

**Additional file 8.** Sensitivity analysis

## Data Availability

Data generated or analyzed during this study are included in this published article and its supplementary information files.

## Abbreviations

COVID-19: Coronavirus disease 2019
WHO: World Health Organization
SARS-CoV-2: Severe acute respiratory syndrome coronavirus-2
LMWH: Low molecular weight heparin
UFH: Unfractionated heparin
P-AC: Prophylactic doses anticoagulation
T-AC: Therapeutic doses anticoagulation
RCTs: Randomized controlled trials
OBs: Observational studies
RR: Risk ratio
Fig.: Figure
DIC: Disseminated intravascular coagulopathy
ICU: Intensive care unit
BMI: Body mass index
IQR: Interquartile range
ARDS: Acute respiratory distress syndrome
QD: Once daily
BID: Twice daily
TID: Thrice daily
VTE: Venous thromboembolism
APTT: Activated partial thromboplastin time
NOS: Newcastle-Ottawa Scale
HIT: Heparin-related thrombocytopenia
