# Supplementary material for: Effect of therapeutic versus prophylactic anticoagulation therapy on clinical outcomes in COVID-19 patients: a systematic review with an updated meta-analysis
Source: Thromb J. 2022 Aug 23;20:47. doi: 10.1186/s12959-022-00408-9 (PMC9395810; doi:10.1186/s12959-022-00408-9)
Supplement: Supplementary file 2 — Additional file 2. PROSPERO Protocol. [file 12959_2022_408_MOESM2_ESM.pdf]

Citation

Huaqin Pan, Hong Duo, Yahui Li, Liang Wei, Yujie Sun, Ziqing Wang. What is the effects of different types and doses of anticoagulants in different COVID-19 patient subgroups. PROSPERO 2021 CRD42021293294 Available from: https://www.crd.york.ac.uk/prospero/display_record.php?ID=CRD42021293294

Review question

Does the use of anticoagulants affect patients with covid-19

Searches

We searched Cochrane COVID-19 register for randomised controlled trials and Observational study without any language restriction in PubMed, EMBASE, the Cochrane Library.

Types of study to be included

Randomised controlled studies (RCTs) and Observational study(OBs).

Condition or domain being studied

Effects of therapeutic and prophylactic doses of heparin on patients with COVID-19.

Participants/population

Patients with COVID-19 who will be included with mild, moderate and severe COVID-19 with any age.

Intervention(s), exposure(s)

Heparin?Heparinoids, Vitamin-K-antagonists, direct anticoagulants. All doses and regimens were eligible.

Comparator(s)/control

Standard of care, placebo or no intervention, other pharmacological and non-pharmacological interventions.

Main outcome(s)

Mortality.

Measures of effect

For continuous outcomes using the same scale we will perform analyses using the mean difference (MD) with 95% confidence intervals (CIs). For continuous outcomes measured with different scales, we will perform analyses using the standardised mean difference (SMD).

We will extract and report hazard ratios (HRs) for time-to-event outcomes.

For dichotomous outcomes, we will perform the analyses using the pooled risk ratio (RR) with a 95% CI.

Additional outcome(s)

- Length of stay in hospital

- Admission to the intensive care unit (ICU)

- minor adverse events and other advers events

- Serious adverse events

- Respiratory support

- Need for invasive mechanical ventilation

- Need for ECMO

- Hospital-acquired infection

Measures of effect

For continuous outcomes using the same scale we will perform analyses using the mean difference (MD) with 95% confidence intervals (CIs). For continuous outcomes measured with different scales, we will perform analyses using the standardised mean difference (SMD).

We will extract and report hazard ratios (HRs) for time-to-event outcomes.

For dichotomous outcomes, we will perform the analyses using the pooled risk ratio (RR) with a 95% CI.

Data extraction (selection and coding)

Two independent researchers will select and extract the data from the included studies. First, the articles will be selected based on the title and abstract. Second, full texts will be evaluated to include or exclude the studies; disagreements will be resolved by consensus and if it is necessary a third researcher will be included. Data regarding authorship, year of publication, patient description, interventions (intervention and control), absolute numbers of each outcome, and follow-up period will be extracted from the studies.

Risk of bias (quality) assessment

The risk of bias for RCT will be used from the Cochrane risk-of-bias (RoB 2) tool and other fundamental elements, being expressed as very serious, serious, or non-serious. The risk of bias assessment will be conducted by two independent reviewers, and in case of disagreement, a third reviewer will be deliberated on the assessment. The quality of the evidence will be extrapolated from the risk of bias and will be described by the Grading of Recommendations Assessment, Development and Evaluation (GRADE) terminology as very low, low, or high.

We will use the RoB 2 Excel tool to implement RoB 2. The full RoB 2 data (e.g., completed Excel tool) will be stored in an online repository.

We will summarize the overall risk of bias for each result (outcome) according to RoB 2 guidance.

Strategy for data synthesis

Categorical outcomes will be expressed by group (intervention and control), the number of events, and calculated risk (in %) for each group (by dividing the number of events by the total number of patients in each group).We will use fixed-effect or random meta-analysis to evaluate the effect of anticoagulants vs. control on the outcomes when those data will be available in at least two RCTs. Effects of meta-analyses will be reported as risk differences (RD) and corresponding 95% CIs; a 95% CI including the number 0 in its range meant that there is no difference in the outcome effect between the intervention and control arms. The use of RD shows the absolute effect size in the meta-analysis when compared with a relative risk (RR) or odds ratio, and this technique can be used when the binary outcome is zero in both study arms. Heterogeneity of effects among studies will be quantified with the I^2 statistic (an $I^2 > 50\%$ means high heterogeneity). For the meta-analysis, we will use the Review Manager software, version 5.4 (RevMan 5; Cochrane Collaboration, Oxford, United Kingdom) or Stata software.

Analysis of subgroups or subsets

We will perform subgroup analyses to calculate RR, MD, or SMD in conjunction with the corresponding CI for each subgroup if statistical heterogeneity is present ($P < 0.01$ for the ?² test of heterogeneity or a different clinical conclusion of 95% CI versus 95% PI).
We will perform subgroup analyses for the following characteristics:
-Subgroups will be considered according the COVID-19 severity

- Risk factors such as age, gender, comorbidities

- Severity of condition (moderate vs. severe disease as defined by the WHO clinical progression scale)

-Different dose of anticoagulants

Contact details for further information

Huaqin Pan
phq2012@whu.edu.cn

Organisational affiliation of the review

Department of Critical Care Medicine, Zhongnan Hospital, Wuhan University

Review team members and their organisational affiliations

Dr Huaqin Pan. Department of Critical Care Medicine, Zhongnan Hospital, Wuhan University
Mr Hong Duo. WuHan University
Miss Yahui Li. WuHan University
Mr Liang Wei. WuHan University
Mr Yujie Sun. WuHan University
Miss Ziqing Wang. WuHan University

Type and method of review

Intervention, Meta-analysis, Systematic review

Anticipated or actual start date

30 November 2021

Anticipated completion date

01 February 2022

Funding sources/sponsors

National Natural Science Foundation of China State the funder, grant or award number and the date of award

Conflicts of interest

Language

English

Country

China

Stage of review

Review Ongoing

Subject index terms status

Subject indexing assigned by CRD

Subject index terms

Administration, Oral; Anticoagulants; Atrial Fibrillation; COVID-19; Humans; SARS-CoV-2

Date of registration in PROSPERO

24 November 2021

Date of first submission

24 November 2021

Stage of review at time of this submission

Stage	Started	Completed
Preliminary searches	Yes	No
Piloting of the study selection process	No	No
Formal screening of search results against eligibility criteria	No	No
Data extraction	No	No
Risk of bias (quality) assessment	No	No
Data analysis	No	No

The record owner confirms that the information they have supplied for this submission is accurate and complete and they understand that deliberate provision of inaccurate information or omission of data may be construed as scientific misconduct.
The record owner confirms that they will update the status of the review when it is completed and will add publication details in due course.

Versions

24 November 2021
