# Supplementary material for: Effect of therapeutic versus prophylactic anticoagulation therapy on clinical outcomes in COVID-19 patients: a systematic review with an updated meta-analysis
Source: Thromb J. 2022 Aug 23;20:47. doi: 10.1186/s12959-022-00408-9 (PMC9395810; doi:10.1186/s12959-022-00408-9)
Supplement: Supplementary file 3 — Additional file 3. Search Strategies. [file 12959_2022_408_MOESM3_ESM.docx]

**Search strategy and registered protocol**

| **Search strategy** | |
| --- | --- |
| **PubMed** | For **PubMed**, the search used was:  (((("Anticoagulants"[Mesh]) OR ("Anticoagulant Drug" OR "Drug, Anticoagulant" OR "Anticoagulant Agents" OR "Agents, Anticoagulant" OR "Anticoagulation Agents" OR "Agents, Anticoagulation" OR "Anticoagulant Drugs" OR "Drugs, Anticoagulant" OR "Anticoagulant Agent" OR "Agent, Anticoagulant" OR "Anticoagulant" OR "Indirect Thrombin Inhibitors" OR "Thrombin Inhibitors, Indirect")) OR ("Heparin"[Mesh])) OR ("Unfractionated Heparin" OR "Heparin, Unfractionated" OR "Heparinic Acid" OR "Liquaemin" OR "Sodium Heparin" OR "Heparin, Sodium" OR "Heparin Sodium" OR "alpha-Heparin" OR "alpha Heparin")) AND ( ("COVID-19"[Mesh]) OR ("COVID 19" OR "SARS-CoV-2 Infection" OR "Infection, SARS-CoV-2" OR "SARS CoV 2 Infection" OR "SARS-CoV-2 Infections" OR "2019 Novel Coronavirus Disease" OR "2019 Novel Coronavirus Infection" OR "2019-nCoV Disease" OR "2019 nCoV Disease" OR "2019-nCoV Diseases" OR "Disease, 2019-nCoV" OR "COVID-19 Virus Infection" OR "COVID 19 Virus Infection" OR "COVID-19 Virus Infections" OR "Virus Infection, COVID-19" OR "Coronavirus Disease 2019" OR "Disease 2019, Coronavirus" OR "Coronavirus Disease-19" OR "Coronavirus Disease 19" OR "Severe Acute Respiratory Syndrome Coronavirus 2 Infection" OR "SARS Coronavirus 2 Infection" OR "COVID-19 Virus Disease" OR "COVID 19 Virus Disease" OR "Disease, COVID-19 Virus" OR "Virus Disease, COVID-19" OR "2019-nCoV Infection" OR "2019 nCoV Infection" OR "2019-nCoV Infections" OR "Infection, 2019-nCoV" OR "COVID19" OR "COVID-19 Pandemic" OR "COVID 19 Pandemic" OR "Pandemic, COVID-19" OR "COVID-19 Pandemics")) |
| **EMBASE** | For **EMBASE**, the search used was:  ('anticoagulants'/exp OR 'anticoagulants' OR 'anticoagulant drug'/exp OR 'anticoagulant drug' OR 'drug, anticoagulant' OR 'anticoagulant agents' OR 'agents, anticoagulant' OR 'anticoagulation agents' OR 'agents, anticoagulation' OR 'anticoagulant drugs' OR 'drugs, anticoagulant' OR 'anticoagulant agent'/exp OR 'anticoagulant agent' OR 'agent, anticoagulant' OR 'anticoagulant'/exp OR 'anticoagulant' OR 'indirect thrombin inhibitors' OR 'thrombin inhibitors, indirect' OR 'heparin'/exp OR 'heparin' OR 'unfractionated heparin'/exp OR 'unfractionated heparin' OR 'heparin, unfractionated' OR 'heparinic acid'/exp OR 'heparinic acid' OR 'liquaemin'/exp OR 'liquaemin' OR 'sodium heparin'/exp OR 'sodium heparin' OR 'heparin, sodium'/exp OR 'heparin, sodium' OR 'heparin sodium'/exp OR 'heparin sodium' OR 'alpha-heparin'/exp OR 'alpha-heparin' OR 'alpha heparin'/exp OR 'alpha heparin') AND ('covid-19'/exp OR 'covid-19' OR 'covid 19'/exp OR 'covid 19' OR 'sars-cov-2 infection'/exp OR 'sars-cov-2 infection' OR 'infection, sars-cov-2' OR 'sars cov 2 infection'/exp OR 'sars cov 2 infection' OR 'sars-cov-2 infections' OR '2019 novel coronavirus disease'/exp OR '2019 novel coronavirus disease' OR '2019 novel coronavirus infection'/exp OR '2019 novel coronavirus infection' OR '2019-ncov disease'/exp OR '2019-ncov disease' OR '2019 ncov disease'/exp OR '2019 ncov disease' OR '2019-ncov diseases' OR 'disease, 2019-ncov' OR 'covid-19 virus infection' OR 'covid 19 virus infection' OR 'covid-19 virus infections' OR 'virus infection, covid-19' OR 'coronavirus disease 2019'/exp OR 'coronavirus disease 2019' OR 'disease 2019, coronavirus' OR 'coronavirus disease-19'/exp OR 'coronavirus disease-19' OR 'coronavirus disease 19'/exp OR 'coronavirus disease 19' OR 'severe acute respiratory syndrome coronavirus 2 infection'/exp OR 'severe acute respiratory syndrome coronavirus 2 infection' OR 'sars coronavirus 2 infection'/exp OR 'sars coronavirus 2 infection' OR 'covid-19 virus disease' OR 'covid 19 virus disease' OR 'disease, covid-19 virus' OR 'virus disease, covid-19' OR '2019-ncov infection'/exp OR '2019-ncov infection' OR '2019 ncov infection'/exp OR '2019 ncov infection' OR '2019-ncov infections' OR 'infection, 2019-ncov' OR 'covid19'/exp OR 'covid19' OR 'covid-19 pandemic' OR 'covid 19 pandemic' OR 'pandemic, covid-19' OR 'covid-19 pandemics') |
| **Web of Science** | For **Web of Science**, the search used was:  **#1:** ALL=(("Anticoagulants") OR ("Anticoagulant Drug" OR "Drug, Anticoagulant" OR "Anticoagulant Agents" OR "Agents, Anticoagulant" OR "Anticoagulation Agents" OR "Agents, Anticoagulation" OR "Anticoagulant Drugs" OR "Drugs, Anticoagulant" OR "Anticoagulant Agent" OR "Agent, Anticoagulant" OR "Anticoagulant" OR "Indirect Thrombin Inhibitors" OR "Thrombin Inhibitors, Indirect"))  **#2:** ALL=(("Heparin") OR ("Unfractionated Heparin" OR "Heparin, Unfractionated" OR "Heparinic Acid" OR "Liquaemin" OR "Sodium Heparin" OR "Heparin, Sodium" OR "Heparin Sodium" OR "alpha-Heparin" OR "alpha Heparin"))  **#3:** ALL=(("COVID-19") OR ("COVID 19" OR "SARS-CoV-2 Infection" OR "Infection, SARS-CoV-2" OR "SARS CoV 2 Infection" OR "SARS-CoV-2 Infections" OR "2019 Novel Coronavirus Disease" OR "2019 Novel Coronavirus Infection" OR "2019-nCoV Disease" OR "2019 nCoV Disease" OR "2019-nCoV Diseases" OR "Disease, 2019-nCoV" OR "COVID-19 Virus Infection" OR "COVID 19 Virus Infection" OR "COVID-19 Virus Infections" OR "Virus Infection, COVID-19" OR "Coronavirus Disease 2019" OR "Disease 2019, Coronavirus" OR "Coronavirus Disease-19" OR "Coronavirus Disease 19" OR "Severe Acute Respiratory Syndrome Coronavirus 2 Infection" OR "SARS Coronavirus 2 Infection" OR "COVID-19 Virus Disease" OR "COVID 19 Virus Disease" OR "Disease, COVID-19 Virus" OR "Virus Disease, COVID-19" OR "2019-nCoV Infection" OR "2019 nCoV Infection" OR "2019-nCoV Infections" OR "Infection, 2019-nCoV" OR "COVID19" OR "COVID-19 Pandemic" OR "COVID 19 Pandemic" OR "Pandemic, COVID-19" OR "COVID-19 Pandemics"))  **#4:** (#1 OR #2) AND #3 |
| **Registered protocol** | |
| The protocol was registered at *https://www.crd.york.ac.uk/prospero/display_record.php?ID=CRD42021293294* | |
