# Supplementary material for: Effect of therapeutic versus prophylactic anticoagulation therapy on clinical outcomes in COVID-19 patients: a systematic review with an updated meta-analysis
Source: Thromb J. 2022 Aug 23;20:47. doi: 10.1186/s12959-022-00408-9 (PMC9395810; doi:10.1186/s12959-022-00408-9)
Supplement: Supplementary file 4 — Additional file 4. PICOs criteria for inclusion and exclusion of studies. [file 12959_2022_408_MOESM4_ESM.docx]

***PICOs* criteria *for inclusion and exclusion of studies***

| Item | Inclusion criteria | Exclusion criteria |
| --- | --- | --- |
| Population | ≥18 years with COVID-19 patients, included both outpatients and inpatients | Non-COVID-19 patients, and < 18 years with COVID-19 patients |
| Intervention | Therapeutic anticoagulation | Other non-anticoagulant drugs such as antiplatelet drug |
| Comparator | Prophylactic anticoagulation | Not therapeutic anticoagulation vs prophylactic anticoagulation, example therapeutic anticoagulation vs placebo |
| Outcome | At least one of outcomes (mortality, major bleeding or thromboembolism) reported | None of outcomes (mortality, major bleeding or thromboembolism) reported |
| Study design | Randomized control trials and Observational studies | Case reports, reviews, editorials, commentaries, practice guidelines, meeting reports, and only abstract form |

Abbreviations:

PICOs: Population, intervention, comparator, outcome and study design; COVID-19: Coronavirus disease 2019
