# Supplementary material for: Effect of therapeutic versus prophylactic anticoagulation therapy on clinical outcomes in COVID-19 patients: a systematic review with an updated meta-analysis
Source: Thromb J. 2022 Aug 23;20:47. doi: 10.1186/s12959-022-00408-9 (PMC9395810; doi:10.1186/s12959-022-00408-9)
Supplement: Supplementary file 5 — Additional file 5. Newcastle-Ottawa Scale. [file 12959_2022_408_MOESM5_ESM.docx]

**Quality analysis of observational studies by Newcastle–Ottawa Scales**

| Study | Selection | | | | Comparability | Outcomes | | | Quality scores |
| --- | --- | --- | --- | --- | --- | --- | --- | --- | --- |
| Author-year | Representativeness of the expose cohort | Selection of the non- exposed cohort | Ascertainment  Of exposure to implants | Demonstration that outcome of interest was not present at start of study | Comparison | Methods of measuring outcomes | Follow-up time | Incomplement of follow-up |  |
| Aschieri-2021 | ☆ | ☆ | ☆ | ☆ | ☆ | ☆ | ☆ | N | 7 |
| Elmelhat-2020 | ☆ | ☆ | ☆ | ☆ | ☆ | ☆ | ☆ | ☆ | 8 |
| Gonzalez-Porras-2021 | ☆ | ☆ | ☆ | ☆ | ☆ | ☆ | N | ☆ | 7 |
| Hamad-2021 | ☆ | ☆ | ☆ | ☆ | ☆☆ | ☆ | ☆ | ☆ | 9 |
| Helms-2021 | ☆ | ☆ | ☆ | ☆ | ☆ | ☆ | ☆ | ☆ | 8 |
| Ionescu-2020 | ☆ | ☆ | ☆ | ☆ | ☆ | ☆ | ☆ | ☆ | 8 |
| Matli-2021 | ☆ | ☆ | ☆ | ☆ | ☆ | ☆ | ☆ | ☆ | 8 |
| Mennuni-2021 | ☆ | ☆ | ☆ | ☆ | ☆ | ☆ | ☆ | ☆ | 8 |
| Takayama-2021 | ☆ | ☆ | ☆ | ☆ | ☆ | ☆ | ☆ | ☆ | 8 |
| Yu-2021 | ☆ | ☆ | ☆ | ☆ | ☆ | ☆ | ☆ | ☆ | 8 |
| Jasmeet 2020 | ☆ | ☆ | ☆ | ☆ | ☆ | ☆ | ☆ | ☆ | 8 |
| Rich Kodama-2020 | ☆ | ☆ | ☆ | N | ☆☆ | ☆ | N | N | 6 |
| Ilaria Battistoni-2021 | ☆ | ☆ | ☆ | ☆ | ☆ | ☆ | ☆ | N | 7 |
| Di Castelnuovo-2021 | ☆ | ☆ | ☆ | ☆ | ☆☆ | ☆ | ☆ | ☆ | 9 |
| Martinelli-2020 | ☆ | ☆ | ☆ | ☆ | ☆ | ☆ | ☆ | N | 7 |
| Rich Kodama-2020 | ☆ | ☆ | N | ☆ | ☆ | N | ☆ | ☆ | 6 |
| Jasmeet Kaur-2020 | ☆ | ☆ | N | ☆ | ☆ | N | ☆ | N | 5 |
| Kadir Canoglu-2020 | ☆ | ☆ | ☆ | N | ☆ | N | N | ☆ | 5 |

Abbreviations:

N: It is not described in the article.
