# Supplementary material for: Effect of therapeutic versus prophylactic anticoagulation therapy on clinical outcomes in COVID-19 patients: a systematic review with an updated meta-analysis
Source: Thromb J. 2022 Aug 23;20:47. doi: 10.1186/s12959-022-00408-9 (PMC9395810; doi:10.1186/s12959-022-00408-9)
Supplement: Supplementary file 6 — Additional file 6: Table S1. Patient Baseline Characteristics in the 11 RCTs. Table S2. Patient Baseline Characteristics in the 17 OBs. [file 12959_2022_408_MOESM6_ESM.docx]

**Table S1 Patient Baseline Characteristics in the 11 RCTs.**

| Author-year | Intervention | Hypertension, n (%) | Cardiovascular disease, n (%) | Diabetes mellitus, n (%) | Chronic kidney disease, n (%) | Chronic pulmonary disease, n (%) | Chronic liver disease, n (%) | History of smoking, n (%) | D-dimer, median (IQR), mg/L | Platelet count,  median (IQR),  x10^9^/L | |
| --- | --- | --- | --- | --- | --- | --- | --- | --- | --- | --- | --- |
| Spyropoulos-2021 | TA | 81 (62.8) | 7 (5.4) | 51 (39.8) | 5 (3.9) | 9 (7.0) | 2 (1.6) | / | 3.8±6.2^a^ | 287.7±119.8^a^ |  |
|  | PA | 70 (56.9) | 11 (8.9) | 43 (34.7) | 4 (3.2) | 8 (6.5) | 1 (0.8) | / | 3.2±5.4^a^ | 269.7±108.2^a^ |  |
| Lemos-2020 | TA | 4 (40) | 1 (10) | 4 (40) | / | / | / | / | 4.2  (2.0-6.4) | 203.7±60.5^a^ |  |
|  | PA | 3 (30) | 1 (10) | 3 (30) | / | / | / | / | 3.4  (1.3-5.5) | 243.8±48.6^a^ |  |
| Goligher-2021 | TA | / | 44 (8.4) | 171 (31.9) | 58 (11.4) | 129 (25.0) | 6 (1.2) | / | 8.2  (4.3-1.7) | 247  (190-317) |  |
|  | PA | / | 45 (8.1) | 191 (33.7) | 43 (8.3) | 129 (24) | 3 (0.5) | / | 0.9  (0.4-1.8) | 244  (182-312) |  |
| Lawler-2021 | TA | 546 (53.4) | 123 (10.6) | 352 (29.8) | 83 (7.1) | 249 (22.0) | / | / | 0.9  (0.7-1.5) | 221  (171-290) |  |
|  | PA | 447 (50.1) | 121 (11.7) | 311 (29.6) | 69 (6.7) | 212 (21.5) | / | / | 1.0  (0.7-1.4) | 218  (173-289) |  |
| Connors-2021 | TA | 57 (34.8) | / | 31 (18.9) | / | / | / | 32 (19.5) | / | 248  (198-302) |  |
|  | PA | 66 (40.0) | / | 36 (21.8) | / | / | / | 29 (17.6) | / | 250  (187-295) |  |
| Marcos-Jubilar-2021 | TA | 10 (31.2) | 3 (9.3) | 2 (6.3) | / | 5 (15.6) | / | 16 (50) | 0.8  (0.6-1.1) | / |  |
|  | PA | 12 (36.3) | 1 (3.0) | 3 (9.1) | / | 6 (18.2) | / | 10 (30.3) | 0.8  (0.6-1.0) | / |  |
| Sholzberg-2021 | TA | 108 (47.4) | 16 (7.0) | 83 (36.4) | 20 (8.8) | 36 (15.8) | 5 (2.2) | 5 (2.2) | 1.0  (0.7, 1.5) | 233.7±95.7^a^ |  |
|  | PA | 117 (49.4) | 18 (7.6) | 77 (32.5) | 13 (5.5) | 27 (11.4) | 9 (3.8) | 7 (3.0) | 1.0  (0.7, 1.7) | 237.8±95.3^a^ |  |
| Morici-2021 | TA | 37 (40.7) | / | 13 (14.3) | 0 (0) | / | / | 16 (17.5) | 0.4  (0.2-0.7) | 211  (161-284) |  |
|  | PA | 29 (31.5) | / | 12 (13.0) | 3 (3.3) | / | / | 13 (14.5) | 0.3  (0.2-0.7) | 195  (163-235) |  |
| Sadeghipour-2021 | TA | 131 (48.0) | 45 (16.3) | 82 (29.7) | / | / | / | 35 (12.7) | 1.0  (0.5-3.1) | 239  (183-309) |  |
|  | PA | 118 (41.2) | 33 (11.5) | 73 (25.6) | / | / | / | 21 (7.3) | 0.9  (0.4-2.4) | 230  (173-301) |  |
| Lopes-2021 | TA | 151 (49) | 12 (4) | 83 (27) | / | 7 (2) | / | 56 (18) | / | / |  |
|  | PA | 151 (50) | 16 (5) | 67 (22) | / | 12 (4) | / | 63 (21) | / | / |  |
| Perepu-2021 | TA | 51 (59) | 27 (31) | 30 (34) | / | 20 (23) | / | 35 (40) | 1.6  (1.0- 4.5) | 257  (217- 332) |  |
|  | PA | 53 (62) | 27 (31) | 34 (40) | / | 19 (22) | / | 38 (44) | 1.9  (0.9- 4.0) | 270  (204- 358) |  |

Abbreviations:

IQR: Interquartile range; TA: Therapeutic anticoagulation; PA; Prophylactic anticoagulation.

^a^ Reported as mean ±standard deviation

**Table S2 Patient Baseline Characteristics in the 17 OBs.**

| Author-year | Intervention | Hypertension, n (%) | Cardiovascular disease, n (%) | Diabetes mellitus, n (%) | Chronic kidney disease, n (%) | Chronic pulmonary disease, n (%) | Chronic liver disease, n (%) | History of smoking, n (%) | D-dimer, median (IQR), ng/mL | Platelet count,  median (IQR),  x10^9^/L | | |
| --- | --- | --- | --- | --- | --- | --- | --- | --- | --- | --- | --- | --- |
| Castelnuovo-2021 | TA | / | / | / | / | / | / | / | / | | / | |
|  | PA | / | / | / | / | / | / | / | / | | / | |
| Elmelhat-2020 | TA | 14 (35.9) | 1 (2.6) | 16 (41) | 2 (5.1) | 1 (2.6) | / | 0 | 13  (10-24) | | / | |
|  | PA | 2 (10) | 0 (0) | 7 (35) | 1 (5) | 0 (0) | / | 0 | 7(4-8) | | / | |
| Gonzalez-Porras-2021 | TA | / | / | / | / | / | / | / | 7.5  (4-13) | | 179  (144-235) | |
|  | PA | / | / | / | / | / | / | / | 8 (5-16) | | 191  (146-253) | |
| Hamad-2021 | TA | 12 (41.4) | 2 (6.8) | 14 (48.3) | 0 (0) | / | / | / | 5.4  (3-15.8) | | 385  (308-466) | |
|  | PA | 8 (47.1) | 0 (0) | 5 (29.4) | 0 (0) | / | / | / | 3.8  (3.3-12.7) | | 320  (184-368) | |
| Helms-2021 | TA | / | 33 (46.5) | 18 (25.3) | 11 (15.5) | 8 (11.3) | 1 (1.4) | / | / | | / | |
|  | PA | / | 43 (39.8) | 13 (12.0) | 5 (4.6) | 12 (11.1) | 2 (1.9) | / | / | | / | |
| Martinelli-2020 | TA | / | / | / | / | / | / | / | 1.1  (0.6-2.2) | | 259  (171-350) | |
|  | PA | / | / | / | / | / | / | / | 1.1  (0.6-2.0) | | 253  (185-341) | |
| Battistoni-2021 | TA | / | / | / | / | / | / | / | / | | / | |
|  | PA | / | / | / | / | / | / | / | / | | / | |
| Ionescu-2020 | TA | 606 (60.7) | / | 346 (34.7) | 83 (8.3) | 249 (24.9) | / | 323 (41.4) | / | | / | |
|  | PA | 1086 (51.2) | / | 595 (28.1) | 105 (5.0) | 433 (20.4) | / | 622 (37.8) | / | | / | |
| Kaur-2020 | TA | / | / | / | / | / | / | / | 0.7  (0.4-2.0) | | / | |
|  | PA | / | / | / | / | / | / | / | 0.5  (0.2-1.0) | | / | |
| Canoglu-2020 | TA | / | / | / | / | / | / | / | / | | / | |
|  | PA | / | / | / | / | / | / | / | / | | / | |
| Qin-2021 | TA | / | / | / | / | / | / | / | / | | / | |
|  | PA | / | / | / | / | / | / | / | / | | / | |
| Matli-2021 | TA | / | / | / | 1 (3.2) | / | 1 (3.2) | 9 (29.0) | 4.60±7.28^a^ | | / | |
|  | PA | / | / | / | 3 (5.9) | / | 0 (0) | 14 (27.5) | 1.06±0.81^a^ | | / | |
| Mennuni-2021 | TA | 93 (62.4) | 27 (18.1) | 29 (19.5) | 17 (11.4) | 12 (8.1) | 3 (2.0) | 20 (13.4) | 5.2±13.9^a^ | | 223±97^a^ | |
|  | PA | 152 (53.0) | 41 (14.3) | 66 (23.0) | 51 (17.8) | 35 (12.2) | 10 (3.5) | 40 (13.9) | 4.2±10.2^a^ | | 213±99^a^ | |
| Kodama-2020 | TA | / | / | / | / | / | / | / | 2.3(0.05)^b^ | | / | |
|  | PA | / | / | / | / | / | / | / | 1.3(0.21)^b^ | | / | |
| Jonmarker-2020 | TA | 17 (46.0) | / | / | 0 (0.0) | / | / | / | 1.3  (0.7-2.9) | | / | |
|  | PA | 32 (47.8) | / | / | 6 (9.0) | / | / | / | 1.8  (1.1-8.4) | | / | |
| Takayama-2021 | TA | / | / | / | / | / | / | 15 (45.5) | 3.7  (2.4-7.5) | | / | |
|  | PA | / | / | / | / | / | / | 14 (48.2) | 2.1  (1.3-6.1) | | / | |
| Yu-2021 | TA | 120 (57.4) | / | 94 (45.0) | 24 (11.5) | 34 (16.3) | 9 (5.5) | / | 1.2  (0.4-5.7) | | 248  (194-312) | |
|  | PA | 322 (42.1) | / | 358  (46.9) | 105 (13.7) | 148 (19.4) | 20 (2.6) | / | 0.5  (0.3-0.9) | | 215  (167-275) |  |

Abbreviations:

IQR: Interquartile range; TA: Therapeutic anticoagulation, PA; Prophylactic anticoagulation.

^a^ Reported as mean ±standard deviation

^b^ Reported as median (# of pts)
