# Supplementary material for: Effect of therapeutic versus prophylactic anticoagulation therapy on clinical outcomes in COVID-19 patients: a systematic review with an updated meta-analysis
Source: Thromb J. 2022 Aug 23;20:47. doi: 10.1186/s12959-022-00408-9 (PMC9395810; doi:10.1186/s12959-022-00408-9)

| **Figures of contents** | Page |
| --- | --- |
| Figure S1 Mortality of therapeutic anticoagulation vs prophylactic anticoagulation in RCTs | 2 |
| Figure S2 Mortality of therapeutic anticoagulation vs prophylactic anticoagulation in OBs | 3 |
| Figure S3 Major bleeding of therapeutic anticoagulation vs prophylactic anticoagulation in RCTs | 4 |
| Figure S4 Major bleeding of therapeutic anticoagulation vs prophylactic anticoagulation in OBs | 5 |
| Figure S5 Subgroup analysis of thromboembolism among critically or non-critically ill patients in RCTs | 6 |
| Figure S6 Subgroup analysis of mortality in studies of patients with high d-dimer level | 7 |
| Figure S7 Sensitivity analysis of mortality in OBs | 8 |
| Figure S8 Funnel plot: Mortality of RCTs | 9 |
| Figure S9 Funnel plot: Major bleeding of RCTs | 10 |
| Figure S10 Funnel plot: Mortality of OBs | 11 |
| Figure S11 Funnel plot: Major bleeding of OBs | 12 |

**Figure S1 Mortality of therapeutic anticoagulation vs. prophylactic anticoagulation in RCTs**


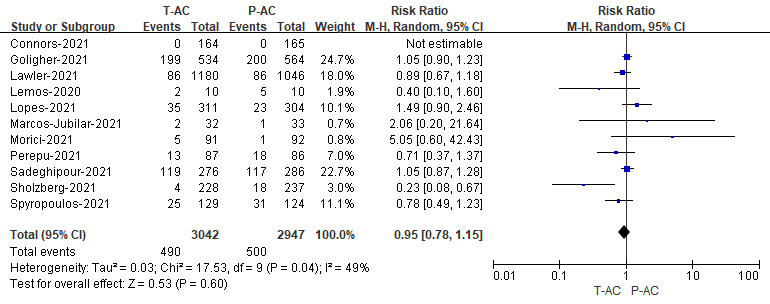


**Figure S2 Mortality of therapeutic anticoagulation vs. prophylactic anticoagulation in OBs**


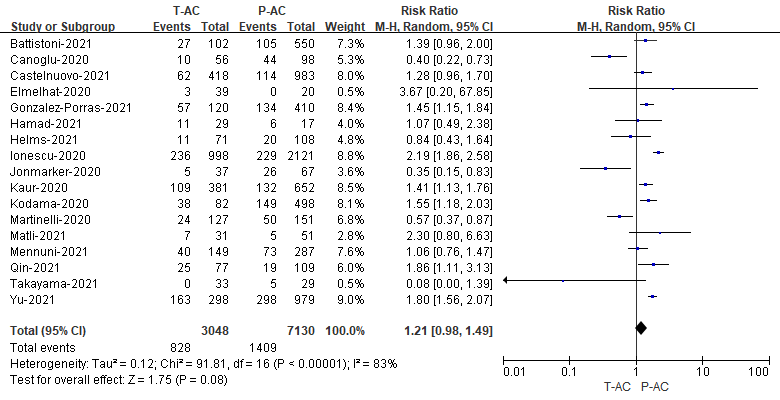


**Figure S3 Major bleeding of therapeutic anticoagulation vs. prophylactic anticoagulation in RCTs**


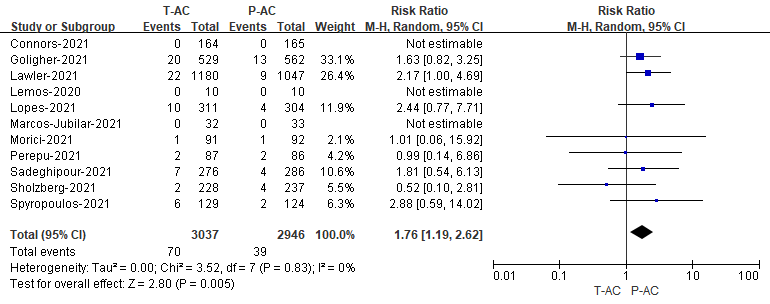


**Figure S4 Major bleeding of therapeutic anticoagulation vs. prophylactic anticoagulation in OBs**


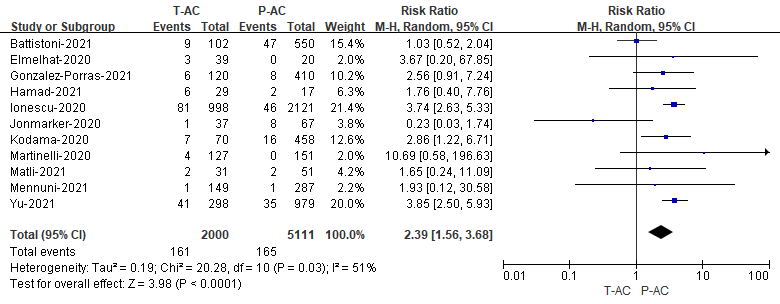


**Figure S5 Subgroup analysis of thromboembolism among critically or non-critically ill patients in RCTs**

**
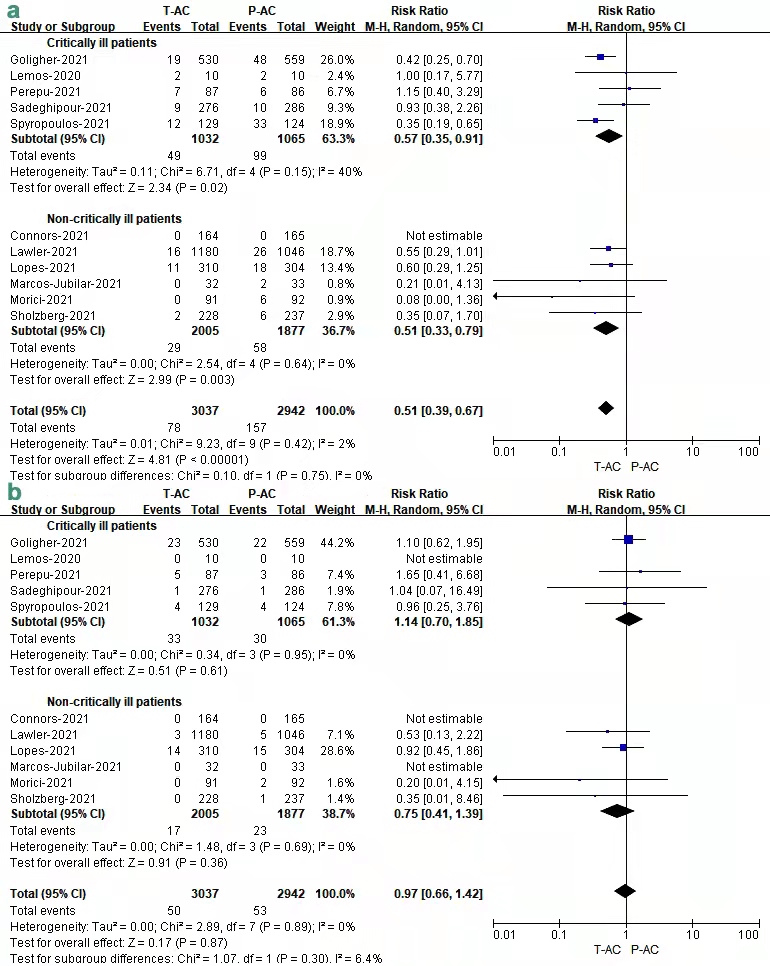
**

**Figure S6 Subgroup analysis of mortality in studies of patients with high d-dimer level.**


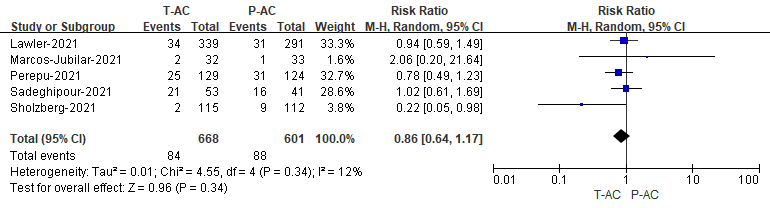


**Figure S7 Sensitivity analysis of mortality in OBs**

**
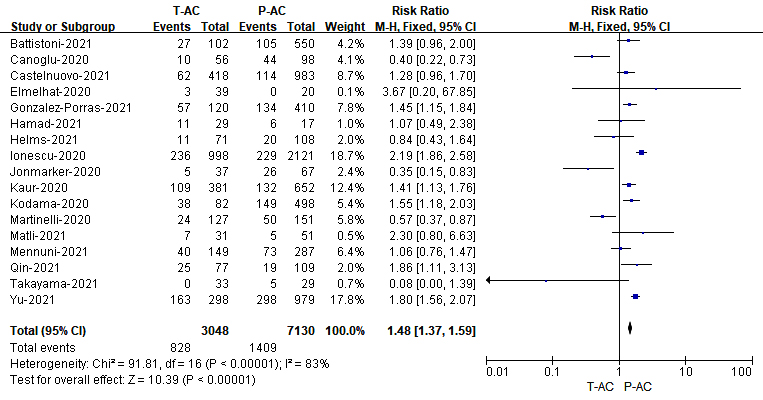
**

**Figure S8 Funnel plot: Mortality of RCTs**


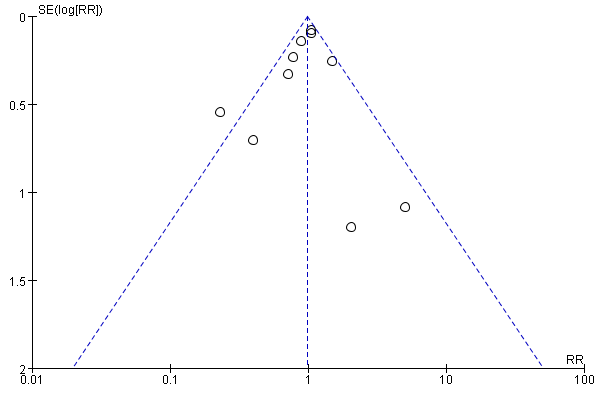


**Figure S9 Funnel plot: Major bleeding of RCTs**


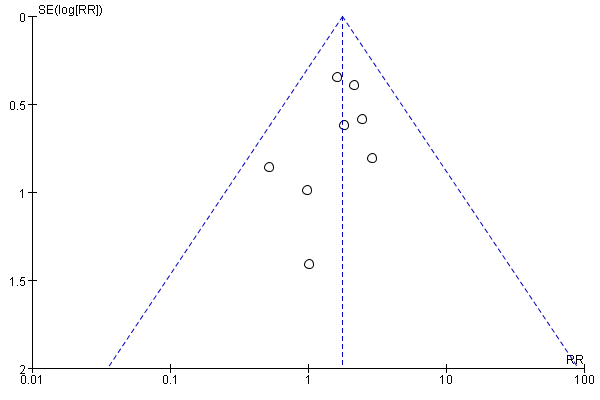


**Figure S10 Funnel plot: Mortality of OBs**


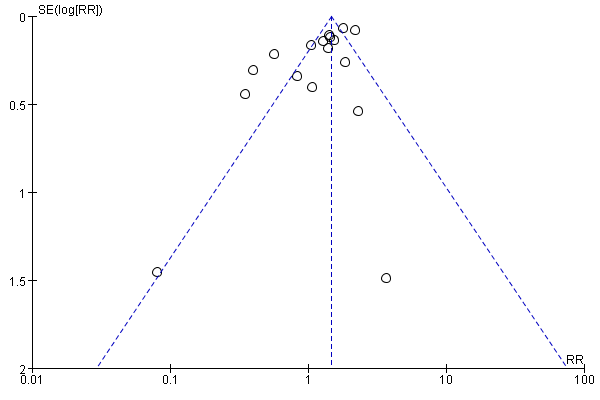


**Figure S11 Funnel plot: Major bleeding of OBs**


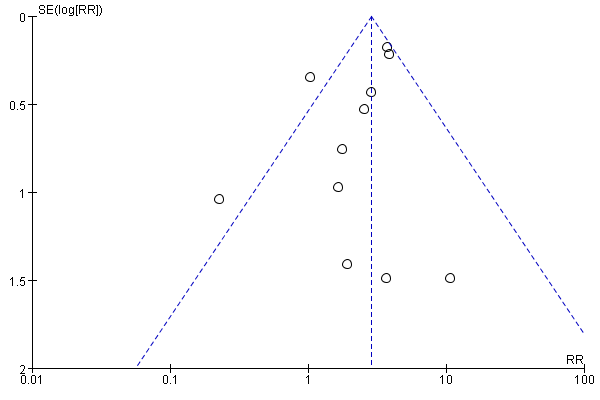

Supplement: Supplementary file 7 — Additional file 7: Fig. S1. Mortality of therapeutic anti-coagulation vs. prophylactic anticoagulation in RCTs. Fig. S2. Major bleeding of therapeutic anti-coagulation vs. prophylactic anticoagulation in RCTs. Fig. S3. Mortality of therapeutic anti-coagulation vs. prophylactic anticoagulation in OBs. Fig. S4. Major bleeding of therapeutic anti-coagulation vs. prophylactic anticoagulation in OBs. Fig. S5. Subgroup analysis of thromboembolism among critically or non-critically ill patients in OBs. Fig. S6. Subgroup analysis of mortality in studies of patients with high d-dimer level. Fig. S7. Sensitivity analysis of mortality in OBs. Fig. S8. Funnel plot: Mortality of RCTs. Fig. S9. Funnel plot: Major bleeding of RCTs. Fig. S10. Funnel plot: Mortality of OBs. Fig. S11. Funnel plot: Major bleeding of OBs. [file 12959_2022_408_MOESM7_ESM.docx]
