# Supplementary material for: Effect of therapeutic versus prophylactic anticoagulation therapy on clinical outcomes in COVID-19 patients: a systematic review with an updated meta-analysis
Source: Thromb J. 2022 Aug 23;20:47. doi: 10.1186/s12959-022-00408-9 (PMC9395810; doi:10.1186/s12959-022-00408-9)
Supplement: Supplementary file 8 — Additional file 8. Sensitivity analysis [file 12959_2022_408_MOESM8_ESM.docx]

***Table S1 Sensitivity analysis***

| Item | Group | Random-effect models | | Fixed-effect models | |
| --- | --- | --- | --- | --- | --- |
|  |  | Heterogeneity | Test for overall effect | Heterogeneity | Test for overall effect |
| Mortality of RCTs | Unprocessed data | Tau² = 0.03;  Chi² = 17.53, df = 9 (*P* = 0.04); I² = 49% | Z = 0.53 (*P* = 0.60) | Chi² = 17.53, df = 9 (*P* = 0.04); I² = 49% | Z = 0.25 (*P* = 0.80) |
|  | The subgroup of critically ill patients | Tau² = 0.00;  Chi² = 4.52, df = 4 (*P* = 0.34); I² = 11% | Z = 0.01 (*P* = 0.99) | Chi² = 4.52, df = 4 (*P* = 0.34); I² = 11% | Z = 0.04 (*P* = 0.97) |
|  | The subgroup of non-critically ill patients | Tau² = 0.29;  Chi² = 12.83, df = 4 (*P* = 0.01); I² = 69% | Z = 0.08 (*P* = 0.93) | Chi² = 12.83, df = 4 (*P* = 0.01); I² = 69% | Z = 0.48 (*P* = 0.63) |
| Major bleeding of RCTs | Unprocessed data | Tau² = 0.00;  Chi² = 3.52, df = 7 (*P* = 0.83); I² = 0% | Z = 2.80 (*P* = 0.005) | Chi² = 3.52, df = 7 (*P* = 0.83); I² = 0% | Z = 2.88 (*P* = 0.004) |
|  | The subgroup of critically ill patients | Tau² = 0.00;  Chi² = 0.75, df = 3 (*P* = 0.86); I² = 0% | Z = 1.96 (*P* = 0.05) | Chi² = 0.75, df = 3 (*P* = 0.86); I² = 0% | Z = 2.01 (*P* = 0.04) |
|  | The subgroup of non-critically ill patients | Tau² = 0.00;  Chi² = 2.74, df = 3 (*P* = 0.43); I² = 0% | Z = 2.01 (*P* = 0.04) | Chi² = 2.74, df = 3 (*P* = 0.43); I² = 0% | Z = 2.06 (*P* = 0.04) |
| Mortality of OBs | Unprocessed data | Tau² = 0.12;  Chi² = 91.81, df = 16 (*P* < 0.00001); I² = 83% | Z = 1.75 (*P* = 0.08) | Chi² = 91.81, df = 16 (*P* < 0.00001); I² = 83% | Z = 10.39 (*P* < 0.00001) |
|  | The subgroup of critically ill patients | Tau² = 0.10;  Chi² = 9.80, df = 6 (*P* = 0.13); I² = 39% | Z = 2.69 (*P* = 0.007) | Chi² = 9.80, df = 6 (*P* = 0.13); I² = 39% | Z = 4.38 (*P* < 0.0001) |
|  | The subgroup of non-critically ill patients | Tau² = 0.03;  Chi² = 27.40, df = 9 (*P* = 0.001); I² = 67% | Z = 5.84 (*P* < 0.00001) | Chi² = 27.40, df = 9 (*P* = 0.001); I² = 67% | Z = 12.77 (*P* < 0.00001) |
| Major bleeding of OBs | Unprocessed data | Tau² = 0.19;  Chi² = 20.28, df = 10 (*P* = 0.03); I² = 51% | Z = 3.98 (*P* < 0.0001) | Chi² = 20.28, df = 10 (*P* = 0.03); I² = 51% | Z = 9.24 (*P* < 0.00001) |
|  | The subgroup of critically ill patients | Tau² = 1.03;  Chi² = 5.45, df = 3 (*P* = 0.14); I² = 45% | Z = 0.60 (*P* = 0.55) | Chi² = 5.45, df = 3 (*P* = 0.14); I² = 45% | Z = 0.77 (*P* = 0.44) |
|  | The subgroup of non-critically ill patients | Tau² = 0.14;  Chi² = 12.76, df = 6 (*P* = 0.05); I² = 53% | Z = 4.55 (*P* < 0.00001) | Chi² = 12.76, df = 6 (*P* = 0.05); I² = 53% | Z = 9.44 (*P* < 0.00001) |

***Table S2 After conducting leave out sensitivity analysis***

| Item | Group | Random-effect models | | Fixed-effect models | |
| --- | --- | --- | --- | --- | --- |
|  |  | Heterogeneity | Test for overall effect | Heterogeneity | Test for overall effect |
| Mortality of RCTs | The subgroup of critically ill patients | Tau² = 0.00;  Chi² = 2.77, df = 3 (*P* = 0.43); I² = 0 | Z = 0.33 (*P* = 0.74) | Chi² = 2.77, df = 3 (*P* = 0.43); I² = 0% | Z = 0.18 (*P* = 0.86) |
|  | The subgroup of non-critically ill patients | Tau² = 0.83;  Chi² = 8.50, df = 2 (*P* = 0.01); I² = 76% | Z = 0.40 (*P* = 0.69) | Chi² = 8.50, df = 2 (*P* = 0.01); I² = 76% | Z = 1.46 (*P* = 0.14) |
|  | The pooled effect | Tau² = 0.03;  Chi² = 13.15, df = 6 (*P* = 0.04); I² = 54% | Z = 0.85 (*P* = 0.40) | Chi² = 13.15, df = 6 (*P* = 0.04); I² = 54% | Z = 0.63 (*P* = 0.53) |
| Major bleeding of RCTs | The subgroup of critically ill patients | Tau² = 0.00;  Chi² = 0.75, df = 3 (*P* = 0.86); I² = 0% | Z = 1.96 (*P* = 0.05) | Chi² = 0.75, df = 3 (*P* = 0.86); I² = 0% | Z = 2.01 (*P* = 0.04) |
|  | The subgroup of non-critically ill patients | Tau² = 0.13;  Chi² = 2.40, df = 2 (*P* = 0.30); I² = 17% | Z = 0.86 (*P* = 0.39) | Chi² = 2.40, df = 2 (*P* = 0.30); I² = 17% | Z = 1.48 (*P* = 0.14) |
|  | The pooled effect | Chi² = 2.40, df = 2 (*P* = 0.30); I² = 17% | Z = 1.48 (*P* = 0.14) | Chi² = 3.17, df = 6 (*P* = 0.79); I² = 0% | Z = 2.49 (*P* = 0.01) |
